# An updated evaluation of the implementation of the sigmoid take-off landmark 1 year after the official introduction in the Netherlands

**DOI:** 10.1007/s10151-023-02803-4

**Published:** 2023-05-15

**Authors:** S. J. A. Hazen, T. C. Sluckin, K. Horsthuis, D. M. J. Lambregts, R. G. H. Beets-Tan, P. J. Tanis, M. Kusters, the Dutch Sigmoid Take-off Research Group, M. Ankersmit, R. R. Bahadoer, I. S. Bakker, F. Bangert, R. M. Barendse, E. Barsom, W. A. Bemelman, K. van den Berg, S. H. de Bie, R. D. Blok, F. C. den Boer, E.-J. G. Boerma, L. S. F. Boogerd, W. A. A. Borstlap, S. J. Braak, J. W. Bradshaw, A. T. A. Brandsma, A. J. A. Bremers, H. J. F. Brenkman, S. W. van der Burg, T. A. Burghgraef, D. W. G. ten Cate, S. H. E. M. Clermonts, L. P. J. Cobben, R. R. J. Coebergh van den Braak, E. C. J. Consten, M. Corver, R. M. P. H. Crolla, S. Curutchet, A. D. van Dalsen, M. Decaestecker, E. B. Deerenberg, E. N. Dekker, T. Derksen, S. van Dijk, A. M. Dinaux, M. Ditzel, E. Dokter, K. Dogan, P. G. Doornebosch, M. C. van Dorth-Rombouts, K. M. A. Dreuning, L. S. E. van Egdom, S. van Elderen, A. M. L. H. Emmen, A. C. van Erp, J. A. van Essen, E. A. Feitsma, S. S. Feshtali, B. Frietman, E. J. B. Furnee, A. M. van Geel, T. H. Geerdink, R. Geitenbeek, A. A. W. Geloven, A. Gerritsen, M. Ghasemi, H. Gielkens, L. Goense, K. M. Govaert, J. A. Govaert, S. Graus, E. J. de Groof, A. A. J. Grüter, R. J. de Haas, P. J. Haasnoot, N. A. G. Hakkenbrak, V. Heesink, S. Hendrickx, S. van den Hoek, E. J. R. J. van der Hoeven, A. Hogewoning, C. R. C. Hogewoning, R. Hompes, A. A. M. Huiberts, J. Jansen, N. Janssen, J. Jonkers, C. de Jonge, D. Jou-Valencia, E. Kaçmaz, D. D. Kamphuis, S. Kanters, I. Kappers, B. Keizers, S. H. J. Ketelaers, M. R. Ketting, S. I. Kreisel, P. A. M. Kint, E. Knöps, S. van Koeverden, S. Kok, J. L. M. Konsten, V. N. N. Kornmann, F. I. de Korte, R. T. J. Kortekaas, A. A. J. M. Kramer-van Tilborg, J. Krdzalic, P. Krielen, L. F. Kroese, B. Lamme, T. Lettinga, A. S. van Lieshout, M. S. de Lijster, F. Logeman, S. A. I. Loggers, J. Luttikhold, T. M. Mackay, M. S. Marsman, M. H. Martens, M. G. Mentink, D. J. L. de Mey, J. Moelker-Galuzina, E. Moltzer, E. J. Mulder, G. D. Musters, J. Nederend, S. Nell, L. C. F. de Nes, J. F. Nieuwenhuis, J. Nonner, B. J. Noordman, S. Nordkamp, S. A. Oei, P. B. Olthof, I. Paulusma, K. C. M. J. Peeters, Z. Pironet, J. D. J. Plate, F. B. Poelmann, I. G. M. Poodt, Z. Popal, L. A. E. Posma, J. F. Prette, A. Pronk, S. M. Qaderi, C. A. L. de Raaff, J. M. van Rees, B. M. M. Reiber, R.-J. Renger, A. J. M. Rombouts, M. de Roos, J. Rothbarth, M. E. van der Sande, B. E. Schaafsma, R. A. Schasfoort, M. M. Scheurkogel, A. Schmid, P. M. E. Schuivens, A. Şekercan, M. van der Sluis, B. P. Smalbroek, L. J. H. Smits, M. N. Sosef, E. J. Spillenaar Bilgen, E. J. A. Steller, J. H. M. B. Stoot, M. Takkenberg, K. Talboom, A. K. Talsma, S. J. D. Temmink, M. Tenhagen, J. Tielbeek, G. F. A. J. B. van Tilborg, G. Y. M. The, D. van Trier, S. A. M. Troquay, J. B. Tuynman, M. J. M. van der Valk, C. J. Veeken, S. L. van Veldhuisen, C. J. Verberne, W. M. Verduin, T. Verhagen, M. Vermaas, V. M. T. van Verschuer, M. Verseveld, G. H. E. J. Vijgen, R. F. A. Vliegen, S. Voets, C. L. A. Vogelij, J. M. Vogten, N. A. Volkers, F. E. E. de Vries, M. de Vries, B. S. T. van Vugt, S. Wang, D. K. Wasowicz, K. Wienholts, J. A. Wegdam, T. J. Weijs, P. P. van Westerveld, H. L. van Westreenen, A. G. Wijma, J. H. W. de Wilt, V. van Woerden, N. Wolfhagen, S. van der Wolk, K. van der Wulp, J. M. Wybenga, E. S. van der Zaag, B. Zamaray, H. J. A. Zandvoort, D. van der Zee, A. Zeilstra, K. J. Zheng, F. M. Zijta, E. S. Zwanenburg

**Affiliations:** 1 Department of Surgery, Amsterdam University Medical Centers, Vrije Universiteit Amsterdam, De Boelelaan 1117, P.O. Box 7057, 1007 MB Amsterdam, the Netherlands; 2 Cancer Center Amsterdam, Treatment and Quality of Life, Amsterdam, The Netherlands; 3 Cancer Center Amsterdam, Imaging and Biomarkers, Amsterdam, The Netherlands; 4 Radiology, Amsterdam UMC, Vrije Universiteit Amsterdam, De Boelelaan 1117, Amsterdam, The Netherlands; 5 Radiology, The Netherlands Cancer Institute, Amsterdam, The Netherlands; 6 GROW School of Oncology and Developmental Biology, University of Maastricht, Maastricht, The Netherlands; 7 Surgery, Amsterdam UMC Location University of Amsterdam, Meibergdreef 9, Amsterdam, The Netherlands; 8 Erasmus Medical Center, Surgical Oncology and Gastrointestinal Surgery, Rotterdam, The Netherlands

**Keywords:** Rectal cancer, Sigmoid cancer, Sigmoid take-off, Implementation, Magnetic resonance imaging

## Abstract

**Purpose:**

The definition of rectal cancer based on the sigmoid take-off (STO) was incorporated into the Dutch guideline in 2019, and became mandatory in the national audit from December 2020. This study aimed to evaluate the use of the STO in clinical practice and the added value of online training, stratified for the period before (group A, historical cohort) and after (group B, current cohort) incorporation into the national audit.

**Methods:**

Participants, including radiologists, surgeons, surgical and radiological residents, interns, PhD students, and physician assistants, were asked to complete an online training program, consisting of questionnaires, 20 MRI cases, and a training document. Outcomes were agreement with the expert reference, inter-rater variability, and accuracy before and after the training.

**Results:**

Group A consisted of 86 participants and group B consisted of 114 participants. Familiarity with the STO was higher in group B (76% vs 88%, *p* = 0.027). Its use in multidisciplinary meetings was not significantly higher (50% vs 67%, *p* = 0.237). Agreement with the expert reference was similar for both groups before (79% vs 80%, *p* = 0.423) and after the training (87% vs 87%, *p* = 0.848). Training resulted in significant improvement for both groups in classifying tumors located around the STO (group A, 69–79%; group B, 67–79%, *p* < 0.001).

**Conclusions:**

The results of this study show that after the inclusion of the STO in the mandatory Dutch national audit, the STO was consequently used in only 67% of the represented hospitals. Online training has the potential to improve implementation and unambiguous assessment.

**Supplementary Information:**

The online version contains supplementary material available at 10.1007/s10151-023-02803-4.

## Introduction

The sigmoid take-off (STO) landmark was chosen as the preferred landmark to define the rectum during a two-round Delphi consensus survey in 2019 with international colorectal experts [1]. The aim of this consensus was to achieve more consistency in the diagnosis of rectal and sigmoid cancer and to improve comparability between trial populations and clinical management [2–5]. The STO landmark was immediately incorporated into the updated Dutch colorectal cancer guideline in 2019, thereby defining a tumor with its lower border below the STO as rectal cancer [6].

This guideline update was disseminated in 2019 through a newsletter of the Dutch Association of Coloproctology with only one accompanying MRI example. To evaluate the implementation and to provide additional information, an online STO training program with a survey was created in 2020 [7]. This training was initially created for a national crossover study, to pursue an unambiguous assessment of the STO in this study. The training resulted in a significant improvement in classifying the tumor according to the STO landmark from 53% before the training to 70% agreement with an expert reference after the training for the 86 participants that had completed the training [7].

In December 2020, the new definition was also officially included in the annual mandatory colorectal cancer audit in the Netherlands. From this moment onwards, Dutch hospitals were obligated to use the STO-based definition of rectal cancer for registration of all surgical resections into the audit. Because of the observed added value of the STO training, the training was offered to surgeons, radiologist, residents, and interns across the Netherlands to further contribute to the implementation of the assessment of this radiological landmark.

This study evaluated whether and to what extent the participants who engaged in this second training round (after inclusion in the national audit program in December 2020) were using the STO consequently during multidisciplinary team (MDT) meetings. Furthermore, this study analyzed the performance and effect of training to apply the STO compared to the first published cohort of participants who participated in the training between 2019 and 2020, to evaluate whether the new cohort, who had more time to use it in the daily practice beforehand, had a higher baseline agreement and whether training is still beneficial. In contrast to the data published in 2020 by Hazen et al., which used a three-category score (“above”, “on”, and “below” the STO), the data were dichotomized as “above” (sigmoid cancer) and “on”/“under” (rectal cancer), to be able to evaluate the classification as used for clinical decision making.

## Materials and methods

### Participants

Surgeons, radiologists, surgical and radiology residents, surgical interns, PhD students, and physician assistants were invited to participate. Group A consisted of 86 participants, who were collaborators of the Snapshot rectal cancer 2016 study, and who completed the STO training after the introduction of the STO in the Dutch guidelines, but before its official adoption as an obligatory landmark in the Dutch national auditing program (December 2020). This group is provided as a historic baseline in this study. Group B consisted of 114 different participants that were recruited via an invitation to participate in the training that was disseminated through a newsletter of the Dutch Association of Coloproctology and through the research network of the Snapshot rectal cancer study. The Dutch Association of Coloproctology has approximately 250 members and consists of colorectal surgeons, surgical residents, and PhD students. The number of specialized abdominal radiologists and radiological residents in training is thought to be similar. Group B completed the training after the official adoption of the STO as an obligatory landmark in the Dutch national auditing program (December 2020).

All 200 participants completed the full training. There were an additional 12 participants who completed the survey, but not the training, and these participants were excluded. There was no overlap in group A and B. To evaluate changes in the use and assessment of the STO-based rectal cancer definition after official adoption in the national auditing program, results of group A (that were previously published by Hazen et al. in 2022 [7]) and group B were compared.

### Training

The training consisted of a baseline questionnaire and 20 MRI cases, a training document explaining the rationale of the STO with five examples on how to classify the tumor, and a post-training assessment with a repeated questionnaire and the same 20 MRI cases in a different order. A single sagittal and axial MR image was provided per case in which the lower border of the tumor and the STO were visible. The participants had to classify the tumor as “below”, “on”, or “above” the STO. A more detailed explanation of the training is provided in a previous publication [7]. The training was provided in a PowerPoint format and from 15 October 2021 also through an automatic e-learning platform (Pluvo/Wend BV, Amsterdam, the Netherlands, https://www.pluvo.co). The content and image quality of the PowerPoint format and the e-learning remained equal. The questionnaires contained questions concerning knowledge of the STO. Surgeons and radiologists specifically were also asked about the use of STO during the MDT meetings. The e-learning platform recorded the completion of the training, while the participants who received the PowerPoint format had to confirm that they completed the training document to ensure that the entire training was completed.

### Patient cases

A consensus meeting was organized with the steering committee, which consisted of experienced specialists from the surgical and radiology department, who also participated in the Delphi consensus rounds, in which the MRI images of the 20 cases and the examples in the training were discussed. In this meeting consensus was reached regarding the classification of the tumors and this was used as the expert reference. A mix of distal rectal tumors, mid rectal tumors, and tumors straddling the STO were chosen. Nine cases were clearly located in the rectum (evident) and 11 cases tumors were located around or above the STO (non-evident). All cases were initially (clinically) diagnosed as rectal tumors, but three out of 20 cases were reclassified in consensus as sigmoid tumors according to the STO definition.

### End points

Primary end points derived from the questionnaires were the percentage of participants being familiar with the new definition for rectal cancer and the percentage of hospitals using the STO as the standard definition for each case during MDT meetings. Primary end points from the case-based assessments were the baseline agreement with an expert reference, improvement in agreement after training, and the inter-observer variability. The hypothesis was that group B was more familiar with the new definition and that the baseline agreement would be higher, owing to more exposure in clinical practice.

### Definition of sigmoid take-off

The STO identifies the point where the rectum with its mesorectum is no longer fixated to the sacrum and where the mesocolon becomes mobile. This point can be identified on MRI or CT. On the sagittal view this is the point where the sigmoid colon moves away horizontally from the sacrum and on the axial plane the sigmoid colon starts to move ventrally. When the lower border of the tumor is located on or under the STO, the tumor is classified as a rectal tumor.

### Statistics

Analyses were performed with IBM SPSS Statistics, version 26.00 (IBM Corp). Categorical variables were described with numbers (*n*) and percentages and continuous variables with means with standard deviations (SD) or median with interquartile range (IQR) for non-normally distribution. The chi-squared test and *t* test were used to compare variables between group A and group B. The generalized estimating equation was used to compare the scores before and after the training within the groups. Furthermore, the sensitivity, specificity, positive predictive value (PPV), and the negative predictive value (NPV) were calculated. A *p* value of less than 0.05 was considered statistically significant. The three locations “under”, “on”, and “above” the STO were dichotomized in “above” (sigmoid cancer) and “on”/“under” (rectal cancer) for all analyses, to be able to evaluate the classification as used in daily clinical practice.

### Ethics and approval

Review board approval was not necessary for this study, owing to no patient involvement and anonymized MRI images.

## Results

Group A consists of 86 participants from 45 hospitals and group B consists of 114 participants from 47 hospitals. The first participant started on 2 October 2020 and the last participant finished on 23 February 2022. Baseline characteristics are shown in Table 1. Group A included 15 surgeons (17%) and 19 radiologists (22%). Group B included 40 surgeons (35%) and group B included 19 radiologists (17%). There was no difference in type of hospital.

**Table 1:** Baseline characteristics of both participating cohorts

	Group A	Group B	*p *value
Number of participants	86	114	
Type of participant			< 0.001
Radiologist	19 (22%)	19 (17%)	
Radiology resident	0	8 (7%)	
Surgeon	15 (17%)	40 (35%)	
Surgical resident	20 (23%)	24 (21%)	
Surgical intern	22 (26%)	10 (9%)	
PhD student	5 (6%)	13 (11%)	
Physician assistant	5 (6%)	0	
Type of hospital			0.052
University	13 (15%)	32 (28%)	
Teaching	65 (76%)	68 (60%)	
Non-teaching	8 (9%)	14 (12%)	
Familiar with sigmoid take-off definition			0.027
No	21 (24%)	14 (12%)	
Yes	65 (76%)	99 (88%)	
Use of STO in MDT as the standard definition for rectal cancer^a^			0.237
No (other definitions are used)	3 (9%)	2 (4%)	
Sometimes (variable use of the STO and other definitions)	14 (41%)	17 (30%)	
Yes (the STO is consequently used)	17 (50%)	38 (67%)	

^a^This question was only addressed to the certified specialists among the group (surgeons and radiologists), the answer of one participant in group B was missing

### Familiarity with and use of sigmoid take-off before the start of the training

Participants in group B were more familiar with the STO landmark (76% vs 88%, *p* = 0.027) before the start of the training. For the certified specialists among the group (i.e., the surgeons and radiologists) there was no difference between the two groups (97% vs 97% in groups A and B, respectively, *p* = 1.000). Although STO was more often consequently used during MDT meetings in group B (50% vs 67%) before the start of the training, this was not significantly different (*p* = 0.237). Some hospitals were represented by more than one specialist. When data were analyzed per hospital that was represented by one or more specialists, 13 of the 28 hospitals (46%) did consequently use the STO during their MDT meetings in group A. In group B, 21 of the 38 hospitals (55%) did consequently use the STO. After the training, only one participant (from group B) indicated that they would still not use the STO as the reference for diagnosing rectal cancer. All other participants were already using the STO or planning to use it as the standard definition of rectal cancer to determine the appropriate treatment after the training.

### Agreement with expert reference and improvement after training

The agreement with the expert reference per case can be found in Table 2 and per type of participant in Table 3. The agreement with the expert reference in reclassifying the tumors was equal for both groups at baseline (80% vs 79%; *p* = 0.423) and also the scores after the training were comparable (87% vs 87%; *p* = 0.848). When data were stratified by type of participant, again no significant differences were found between group A and B. The SDs of the scores were smaller for both groups after the training, showing a reduction in the variation. For group A the SD changed from 10 to 5 and for group B from 11 to 5. Non-significant improvement after the training was found for group A (80% vs 87%; *p* = 0.078), but significant improvement was observed in group B (79% vs 87%; *p* = 0.025). When agreement was determined for the non-evident cases only (i.e., difficult cases with tumors near the STO), effects became more apparent and the scores improved from 69% to 79% in group A and from 67% to 79% in group B after the training (*p* < 0.001 for both groups).

**Table 2:** Agreement with expert reference per case before and after the training

Group A		Baseline			After training			Group B		Baseline			After training		
Case	Reference	Rectal (%)	Sigmoid (%)	Agreement (%)	Rectal (%)	Sigmoid (%)	Agreement (%)	Case (%)	Reference	Rectal (%)	Sigmoid (%)	Agreement (%)	Rectal (%)	Sigmoid (%)	Agreement (%)
1	Rectal (E)	98	2	98	98	2	98	1	Rectal (E)	99	1	99	96	4	96
2	Rectal (E)	61	38	61	87	13	87	2	Rectal (E)	70	29	70	94	6	94
3	Rectal (E)	99	1	99	99	1	99	3	Rectal (E)	98	2	98	100	0	100
4	Rectal (N)	61	17	61	91	7	91	4	Rectal (N)	79	20	79	90	10	90
5	Rectal (N)	85	15	85	100	0	100	5	Rectal (N)	77	23	77	96	4	96
6	Rectal (E)	100	0	100	100	0	100	6	Rectal (E)	99	1	99	100	0	100
7	Rectal (N)	78	22	78	92	8	92	7	Rectal (N)	72	27	72	92	8	92
8	Rectal (N)	75	26	75	86	14	86	8	Rectal (N)	75	24	75	98	3	98
9	Rectal (N)	80	21	80	99	1	99	9	Rectal (N)	78	21	78	98	2	98
10	Sigmoid (N)	61	40	40	94	6	6	10	Sigmoid (N)	57	43	43	81	20	20
11	Rectal (E)	93	7	93	98	2	98	11	Rectal (E)	88	11	88	97	3	97
12	Rectal (E)	99	1	99	100	0	100	12	Rectal (E)	94	6	94	100	0	100
13	Rectal (N)	80	20	80	97	4	97	13	Rectal (N)	75	25	75	97	3	97
14	Rectal (E)	92	8	92	92	8	92	14	Rectal (E)	90	11	90	92	8	92
15	Sigmoid (N)	40	61	61	58	42	42	15	Sigmoid (N)	31	69	69	58	42	42
16	Rectal (E)	100	0	100	100	0	100	16	Rectal (E)	98	2	98	100	0	100
17	Rectal (E)	99	1	99	100	0	100	17	Rectal (E)	98	2	98	100	0	100
18	Rectal (N)	65	35	65	98	2	98	18	Rectal (N)	62	38	62	95	5	95
19	Sigmoid (N)	34	66	66	33	66	66	19	Sigmoid (N)	29	70	70	40	59	59
20	Rectal (N)	48	52	48	94	6	94	20	Rectal (N)	37	62	37	84	16	84
Total		77	22	80	91	9	87	Total		75	24	79	90	10	87

*E* evident case, clear rectal tumor, *N* non-evident, straddling the sigmoid take-off

**Table 3:** Agreement with expert reference before and after training stratified by type of participant before (group A) and after (group B) the official introduction of the new definition of rectal cancer

	Score baseline			Score after training		
	Group A	Group B	*p *value	Group A	Group B	*p *value
	Score % (SD)	Score % (SD)		Score % (SD)	Score % (SD)	
Type of participant						
Radiologist	82% (8)	83% (10)	0.555	88% (5)	88% (5)	0.871
Radiology resident	NA	79% (5)	NA	NA	87% (6)	NA
Surgeon	80% (11)	85% (6)	0.411	85% (6)	88% (6)	0.122
Surgical resident	80% (11)	80% (9)	0.997	87% (5)	87% (5)	0.689
Surgical intern	75% (10)	80% (14)	0.325	87% (6)	85% (5)	0.331
PhD student	90% (6)	77% (18)	0.139	92% (3)	89% (6)	0.302
Physician assistant	86% (5)	NA	NA	88% (6)	NA	NA
Total	80% (10)	79% (11)	0.423	87% (5)	87% (5)	0.848

*NA = not applicable*

### Diagnostics

The sensitivity to diagnose rectal cancer (positive outcome) before the training was 85% in group A and 82% in group B. This improved to 96% and 96%, respectively. The specificity to diagnose sigmoid cancer (negative outcome) before the training was 55% in group A and 60% in group B. This decreased to 38% and 40%, respectively. The PPV was 92% before the training and 90% after the training for group A as well as group B. The NPV improved in both groups: from 39% to 63% in group A, and from 37% to 63% in group B.

## Discussion

The results of this study show that the familiarity with the STO-based definition of rectal cancer in the Netherlands before the start of the training has improved from 76% since its initial introduction into the Dutch national guidelines in 2019 to 88% after the official introduction in the mandatory Dutch colorectal cancer audit in 2020. However, despite this higher familiarity (and an extra year of clinical practice), the classification of the tumors according to the STO landmark on MRI, compared to an expert reference, did not show further improvement compared to the previous “baseline” study published following the Dutch guideline updates. Additional targeted training in the assessment of the STO still improved the classification of particularly tumors located around the STO from 67% to 79% with less variation between the observers. Therefore, these results highlight the importance of accompanying training when implementing new guidelines, as unstructured introduction into clinical practice appears to be less effective.

Although the familiarity with the STO improved to 88% in group B (i.e., the group analyzed after implementation of the STO in the Dutch national audit), the reported consistent use of the STO during MDT meetings was only 67%. This is an important finding considering that STO has been adopted as a mandatory landmark in the Dutch clinical guidelines and as an integral part of the Dutch clinical auditing system, this seems to be a disappointingly low number. On a positive note, all participating medical specialists, except one, indicated that they were planning to use the new definition during the MDT meetings after the training. This shows that extra exposure in the form of a training program can encourage specialists to change local practice. Another way to improve assessment and reporting of essential characteristics is by using templates for MRI rectal cancer staging reports. Templates for MRI rectal cancer reports have been increasingly used over the past years and resulted in an increase in the reporting of key elements for clinical decision-making [8–11]. By including the STO into such a template, radiologists will be stimulated to use this classification.

There is often a gap between evidence generated by research and its subsequent implementation into daily clinical practice [12, 13]. A Swedish study evaluated specifically the content of MRI reports on rectal cancer and compared this to evidence-based practice, showing an evident gap in which half of the clinically important imaging information needed for treatment planning was omitted [14]. Especially now we increasingly have online tools available, the time between guideline changes and implementation can be shorted, and the implementation itself can be further improved by the use of online training [15]. Different studies have evaluated the effectiveness of e-learning. A systematic review assessing e-learning for surgical training by Maertens et al. did show greater effectiveness compared to no intervention or non-e-learning interventions in large proportions of the included study [16]. Also for radiological subjects, promising results have been reported. A clear reduction in inter-observer variability was for example seen in a training program in the assessment of lateral lymph nodes on MRI [17] and an online curriculum for surgical trainees on the interpretation of pelvic MRI [18].

Despite increased familiarity with the STO following the Dutch national guideline updates, agreement with an expert reference (before dedicated training) did not improve over time and after the mandatory adoption of the STO into the Dutch clinical auditing system. When the STO-based definition of rectal cancer was included in the updated national guideline, the surgeons were notified through a newsletter with only one MRI picture as an example, which was derived from the original Delphi consensus publication [1]. This information was insufficient, because the Delphi consensus publication only provided a definition for the rectum and not how to apply this specifically to diagnose rectal cancer and discern it from sigmoid cancer. Furthermore, the MRI images used in the Delphi consensus publication were from a case with standard pelvic anatomy without pathology and an extraordinary clear STO. In clinical practice, the exact location of the STO might be more difficult to determine because of variations in anatomy, but also as a result of previous surgical history, presence of tumor, presence of anorectal fluid, and complications such as intussusception causing a shift in anatomy [19].

In the current study, still 13% of the cases were classified incorrectly after the training and this was 21% for cases which were located around the STO. This resulted mostly in an increase in false positives (sigmoid tumors incorrectly diagnosed as rectal tumors) and consequently a decrease in specificity. A recent study which evaluated the reproducibility of the STO by radiologists and surgeons showed at least 80% agreement in only 58–63% of the tumors near the rectosigmoid junction [19]. In this previous study by Bogveradze et al., agreement was considerably better (72%) for more experienced observers, suggesting that training can have an important additional value [19]. However, the “only” 72% agreement for experienced observers does also show that there will always remain difficult cases for which no consensus can be reached [19]. The difficulty of assessing the STO might be one of the reasons why the STO is not consistently used in the MDTs and why other international guidelines have not included this definition yet.

In the current study, improvement in agreement after the training was especially seen for the non-evident cases, which were located around the STO. A second training session specifically focusing on the difficulties and pitfalls can contribute to further improving the assessment of the STO and to further minimize the variability in classifying the tumors according to the STO. When the STO is a proven workable landmark with acceptable variability, other guidelines will be encouraged to use this definition, for which it is essential to provide accompanying training for optimal implementation.

The new definition is expected to have consequences for the treatment of the redefined sigmoid tumors. These tumors will not qualify for neoadjuvant radiotherapy anymore, except cT4b tumors. Furthermore, in case of pathological lymph nodes there would be an indication for adjuvant systemic chemotherapy according to the Dutch guideline. However, the Dutch guideline deviates from other guidelines on this subject, because in the American Society of Colon and Rectal Surgeons (ASCRS) guideline rectal tumors also qualify for adjuvant chemotherapy. The proportion of redefined sigmoid tumors, the consequences for the treatment, and the oncological outcomes of these tumors will be evaluated in another study by the Dutch Snapshot Research Group.

### Limitations

This study has several limitations. Firstly, the participants were provided with two or three images of the MRI, which makes it more difficult to determine the lower border of the tumor and the exact location of the STO compared to investigation of the full MRI. However, only cases were selected in which the STO and the tumor were visible on the two-dimensional images. Secondly, the voluntary character of the training may have caused selection bias regarding participation by invited physicians. Clinicians who are experienced with the STO might be underrepresented, because they do not see an added value of the training, or over-represented, as clinicians who generally do not participate in training programs will also not be reached by voluntary training initiatives and remain inexperienced. Lastly, the gold standard was the expert reference, and considering the previously reported agreement of 72% under expert radiologists [19], the gold standard is relative and it is not possible to obtain an actual valid gold standard for this diagnosis.

## Conclusion

After the official introduction of the STO-based definition of rectal cancer the familiarity with this new definition has increased. However, increased familiarity together with an extra year of experience in the assessment of this landmark in clinical practice did not improve the baseline assessment. Online training still appeared to be beneficial, especially in decreasing variability between the participants, and the results of this study therefore highlight the importance of an accompanying training program in the implementation of a guideline or a new clinical development.

### Supplementary Information

Below is the link to the electronic supplementary material.

Supplementary file1 (DOCX 142 KB)

Supplementary file2 (PDF 6866 KB)

## Data Availability

The data that support the findings of this study are available from the corresponding author, MK, upon reasonable request.
