# Supplementary material for: An updated evaluation of the implementation of the sigmoid take-off landmark 1 year after the official introduction in the Netherlands
Source: Tech Coloproctol. 2023 May 15;27(12):1243–50. doi: 10.1007/s10151-023-02803-4 (PMC10638143; doi:10.1007/s10151-023-02803-4)
Supplement: Supplementary file 1 — Supplementary file1 (DOCX 142 KB) [file 10151_2023_2803_MOESM1_ESM.docx]

**
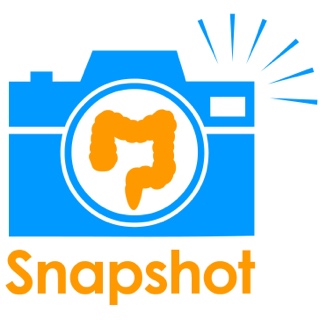

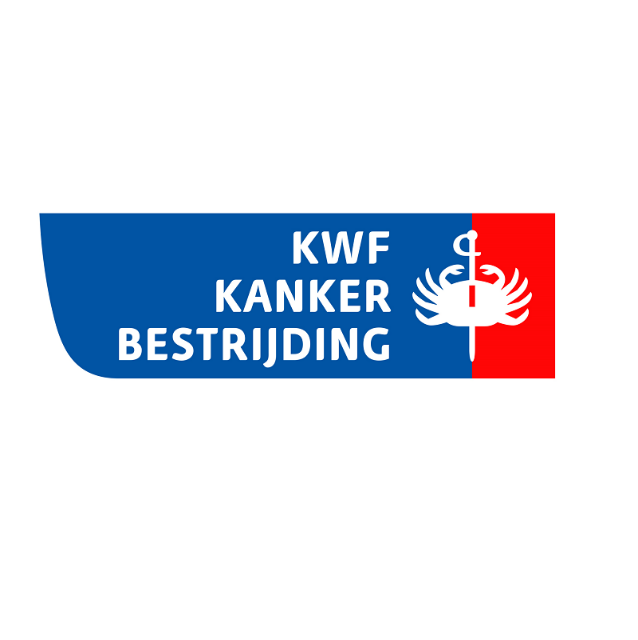

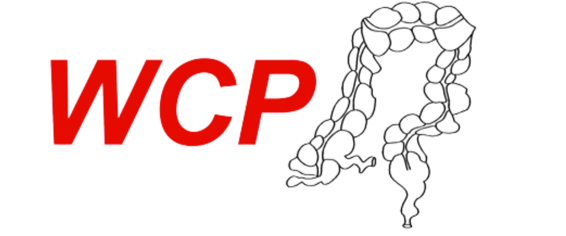
**

**Appendix Ia Questionnaire Sigmoid take-off before the training**

Name:

Hospital:

Date:

Type of participant:

Radiologist
 Radiology resident
 Surgeon
 Surgical resident
 Surgical intern
 PhD-student
 Physician assistant

Before

1. On a scale from 1-10, how do you rate yourself in analyzing a MRI-scan of the rectum?

1 2 3 4 5 6 7 8 9 10

2. Where do you localize the border between the sigmoid and rectum?

3. Have you ever heard of the term “sigmoid take-off”?

Yes

No

3a. If yes, how do you determine the sigmoid take-off?

**Only for surgeons and radiologists:**

4. Is the sigmoid take-off consequently used in the multidisciplinary team meeting in your hospital as the standard border between the sigmoid and rectum, so that tumors above the sigmoid take-off do not receive routinely neoadjuvant radiotherapy anymore?

Yes  No  Variable

4a. If no or variable, could you explain your answer further

4b. If yes, since when is the sigmoid take-off used in the multidisciplinary team meeting?

**Only for radiologists:**

5. How often are you present in the multidisciplinary team meetings in which colorectal cancer patients are discussed (please provide your answer in percentage)


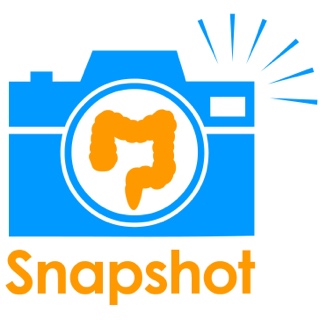

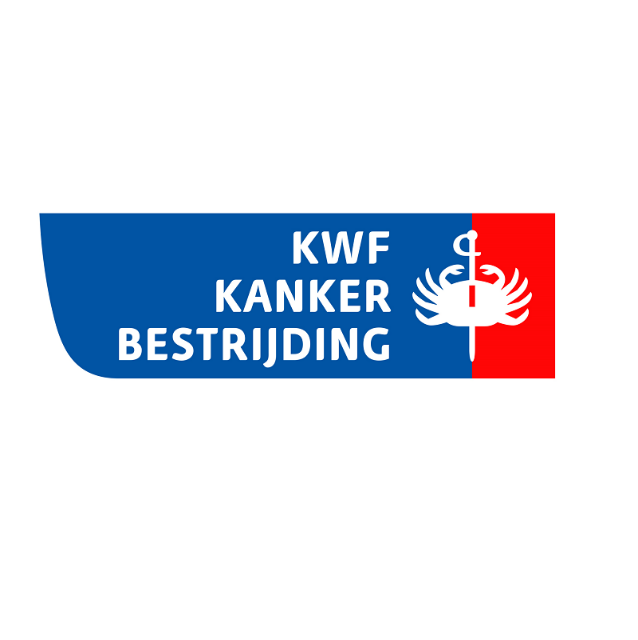

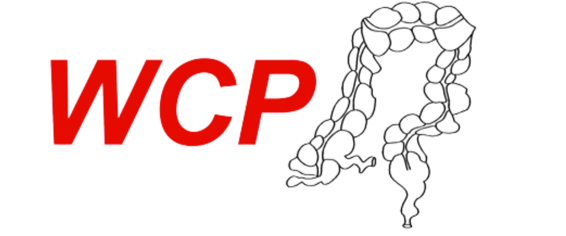


**Appendix Ib Questionnaire Sigmoid take-off after the training**

Name:

Hospital:

Date:

Type of participant:

Radiologist
 Radiology resident
 Surgeon
 Surgical resident
 Surgical intern
 PhD-student
 Physician assistant

1. On a scale from 1 (easy) to 10 (difficult), how difficult did you find it to understand the sigmoid take-off? ?

1 2 3 4 5 6 7 8 9 10

2. Do you find the sigmoid take-off landmark a good landmark to determine the border between the rectum and sigmoid?

Yes

No

2a. If no, which border or level would you rather use?

3. What did you find difficult in determining the sigmoid take-off?

4. What did you find easy in determining the sigmoid take-off?

**Only for surgeons and radiologists:**

5. Are you planning to use the sigmoid take-off as reference for diagnosing rectal cancer?

Yes, we were already using it

Yes, we are planning to use it

No
