# Supplementary material for: An updated evaluation of the implementation of the sigmoid take-off landmark 1 year after the official introduction in the Netherlands
Source: Tech Coloproctol. 2023 May 15;27(12):1243–50. doi: 10.1007/s10151-023-02803-4 (PMC10638143; doi:10.1007/s10151-023-02803-4)

Sigmoid take-off

Patient 1

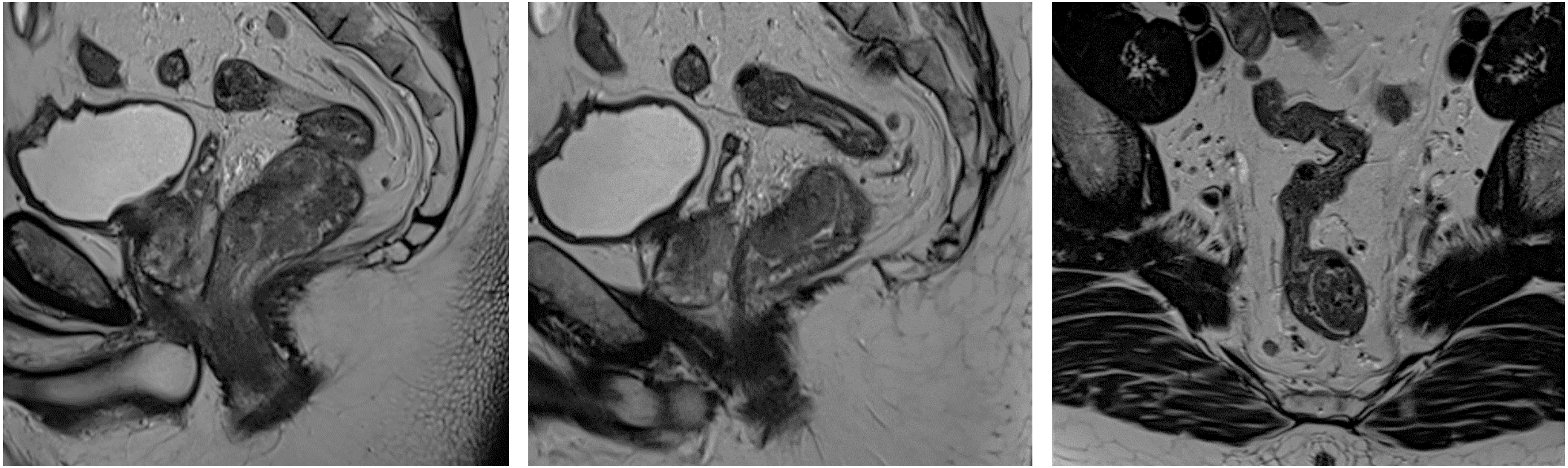

Patient 2

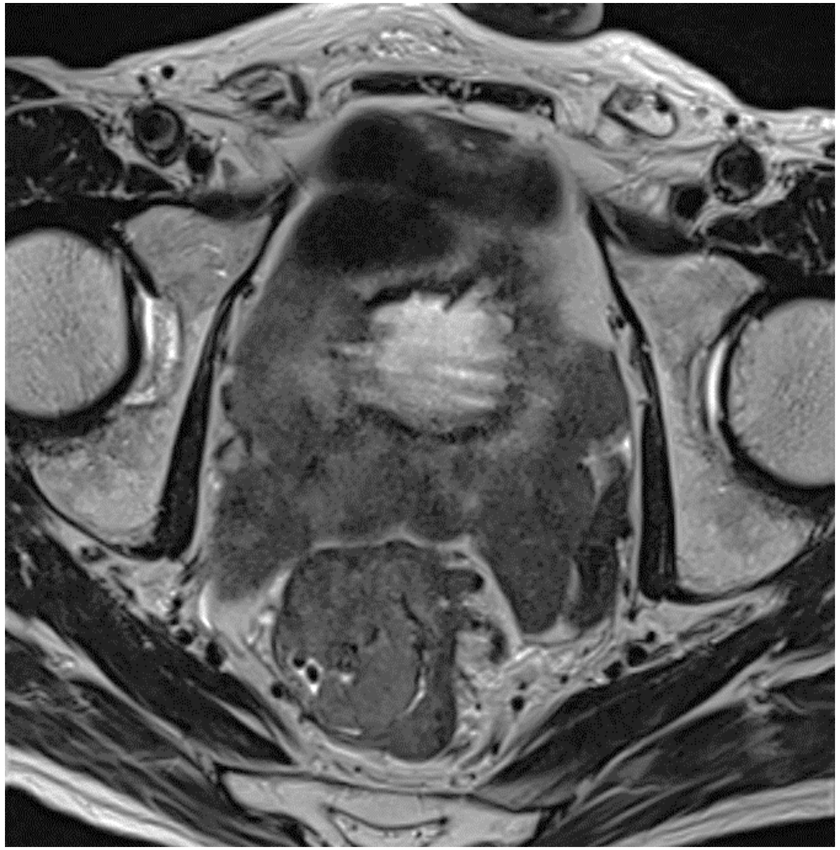

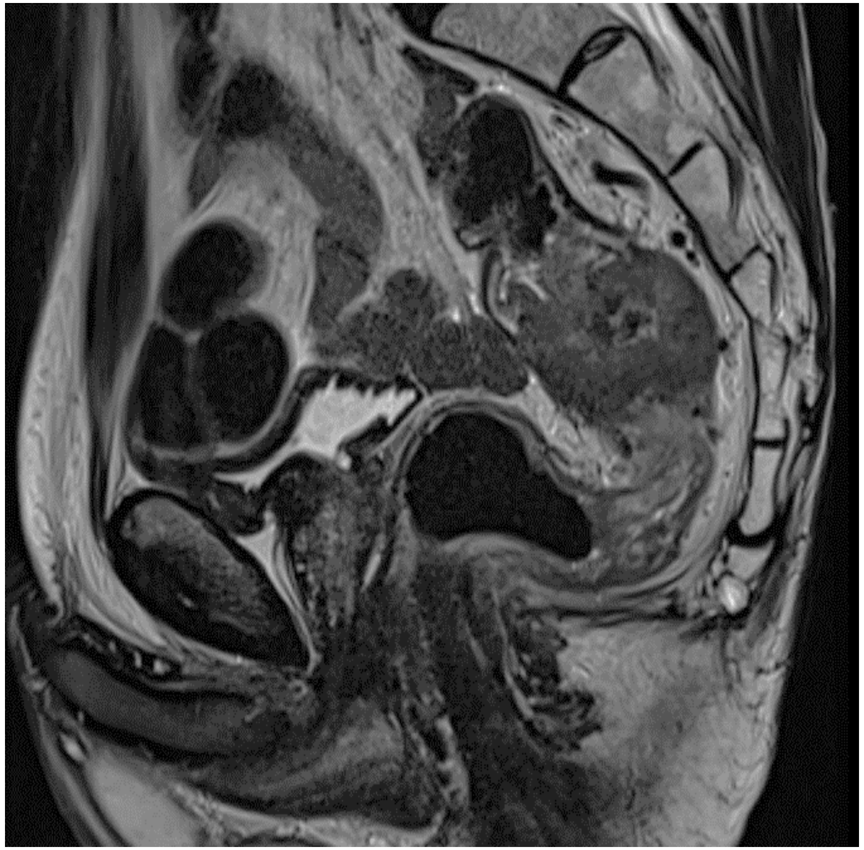

Patient 3

Snapshot

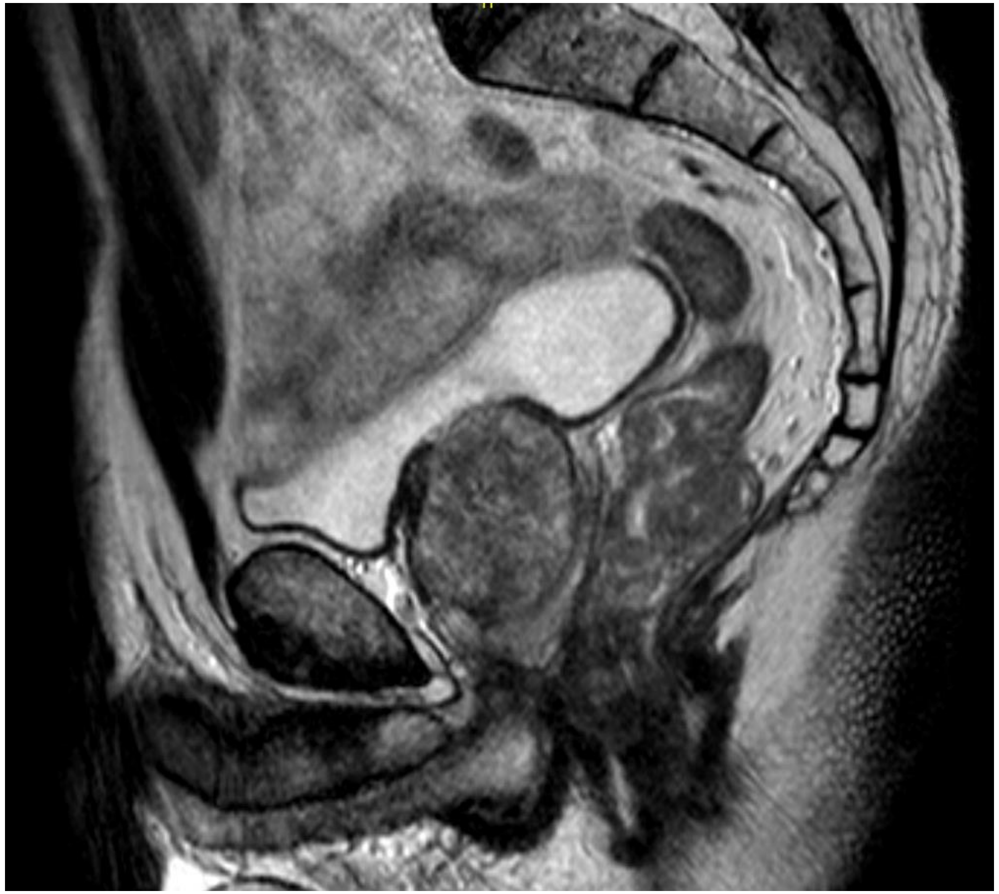

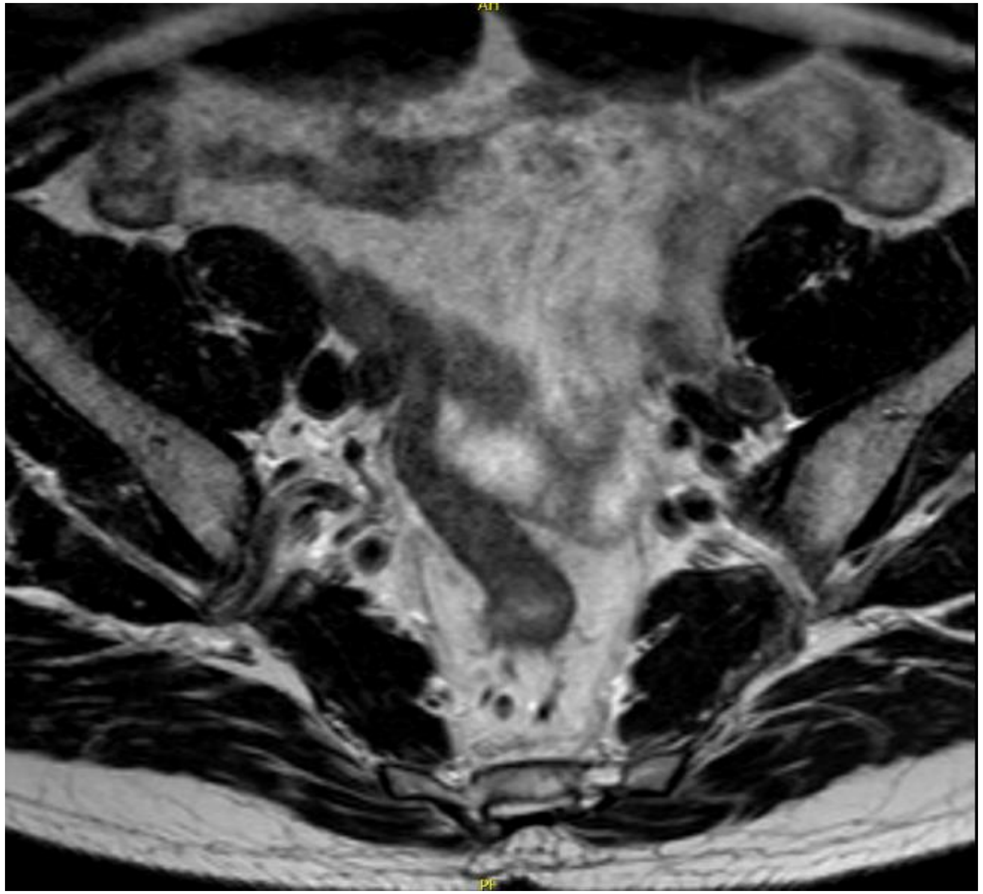

Patient 4

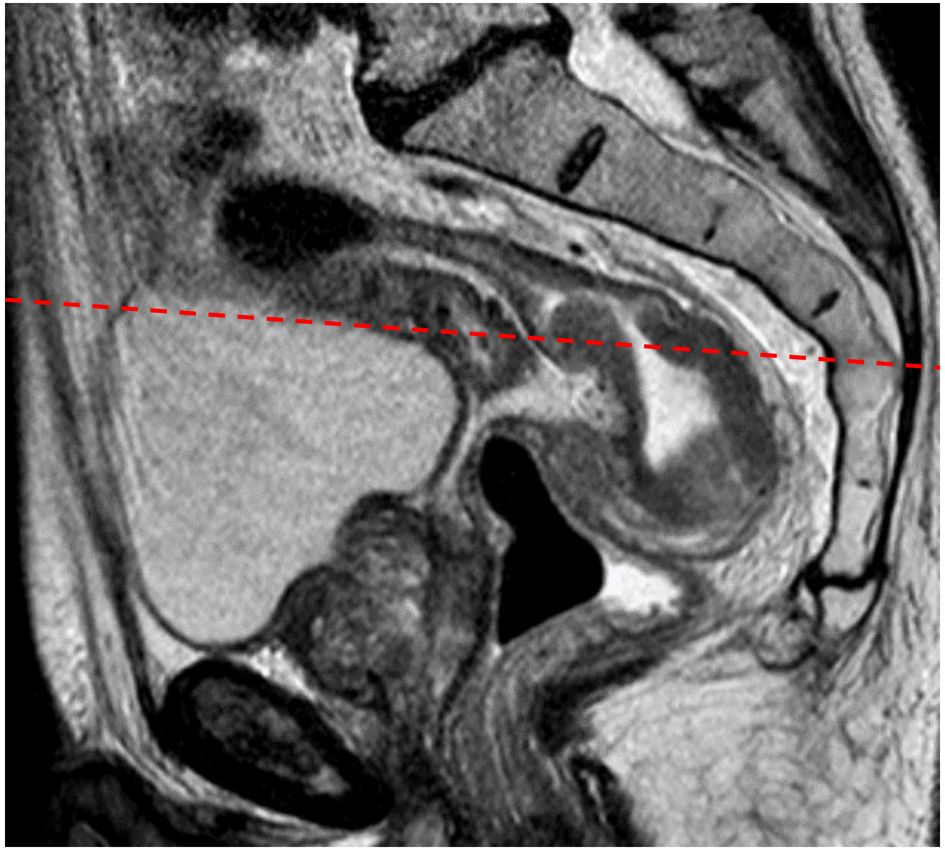

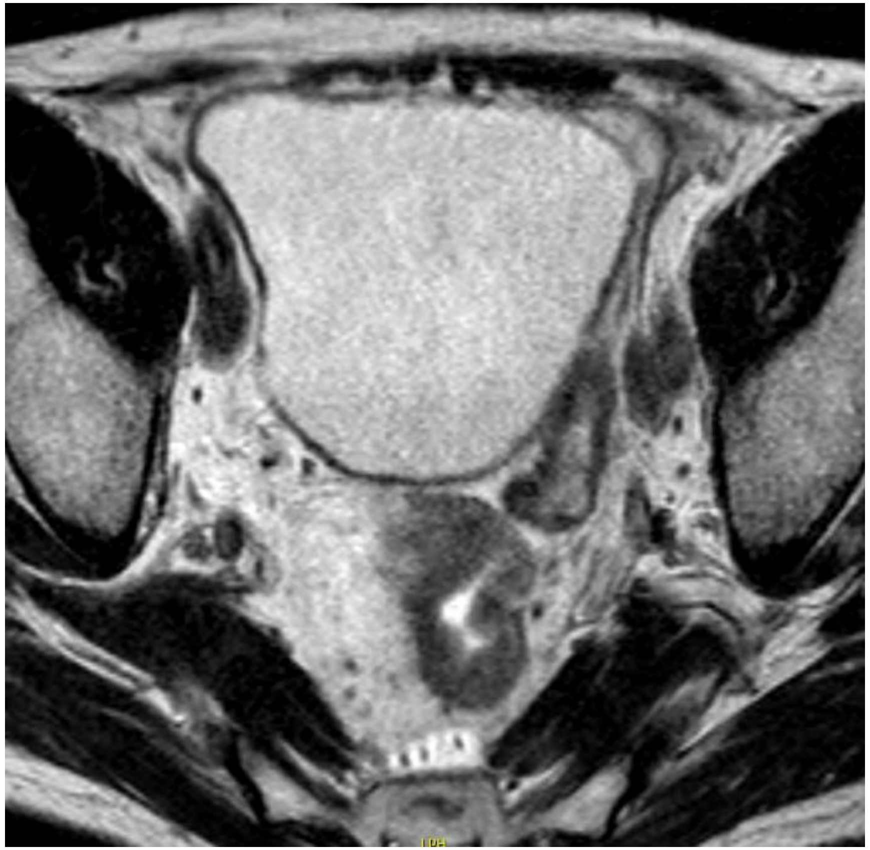

Patient 5

Snapshot

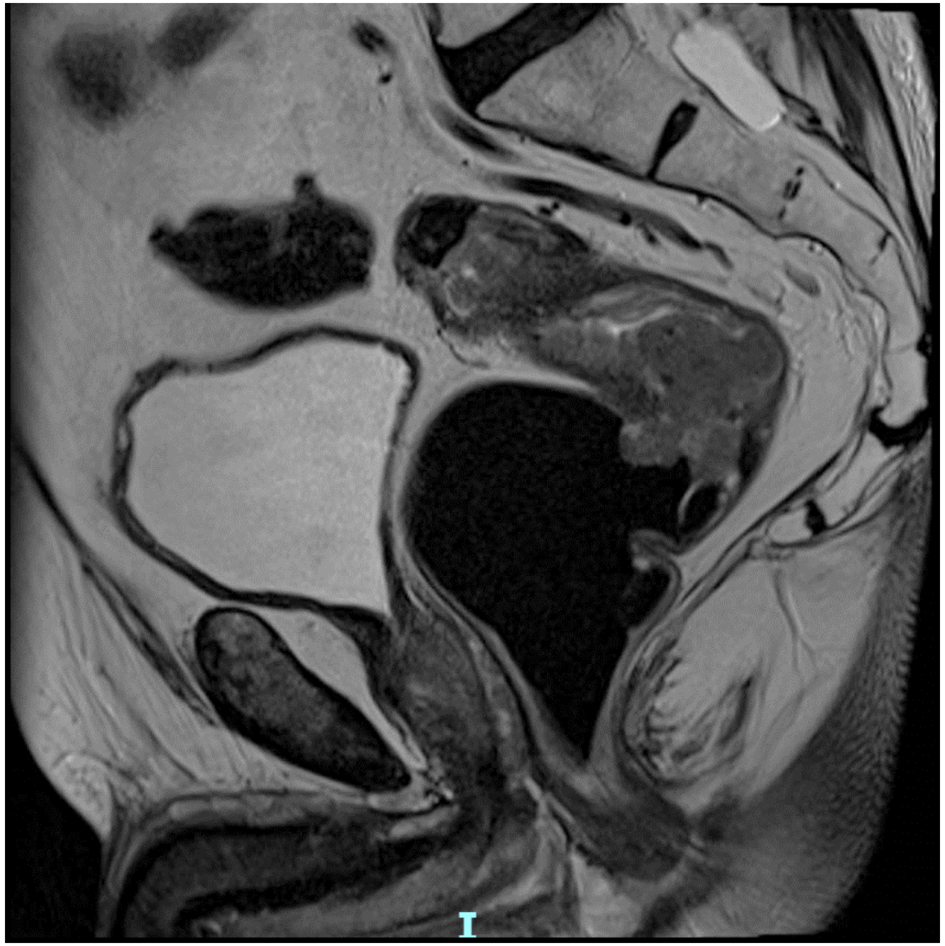

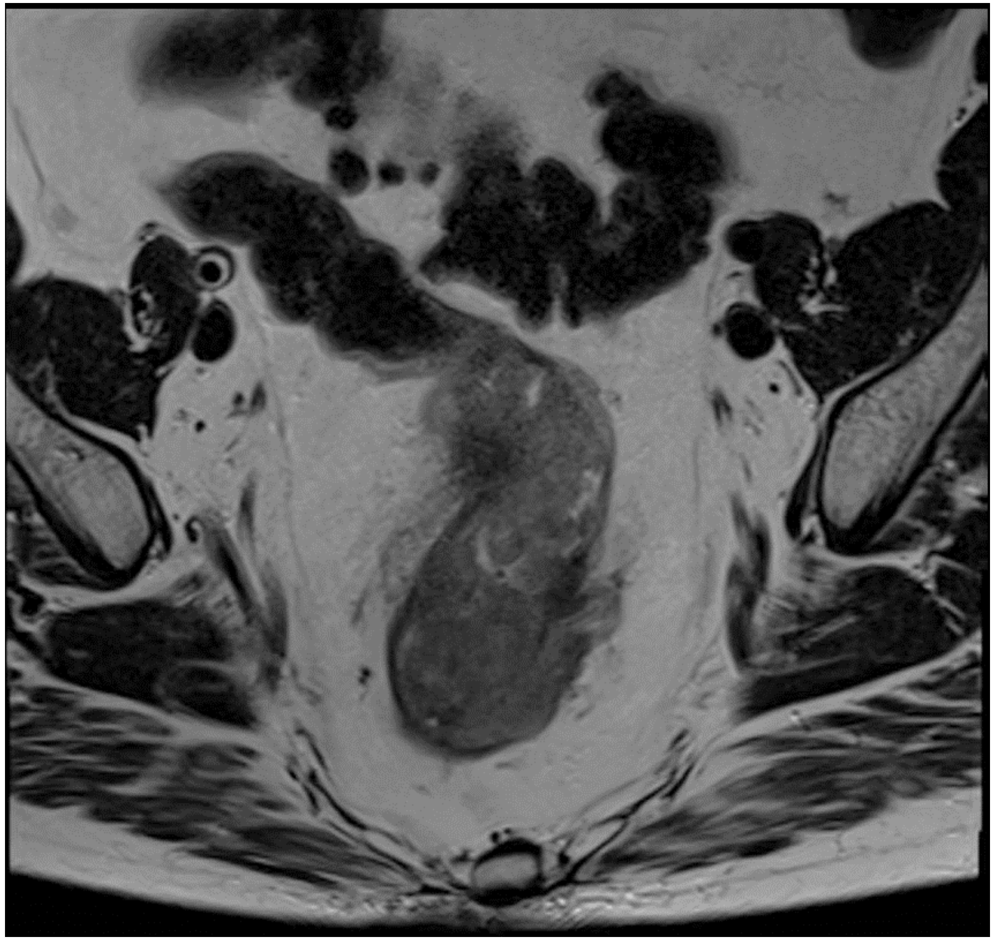

Patient 6

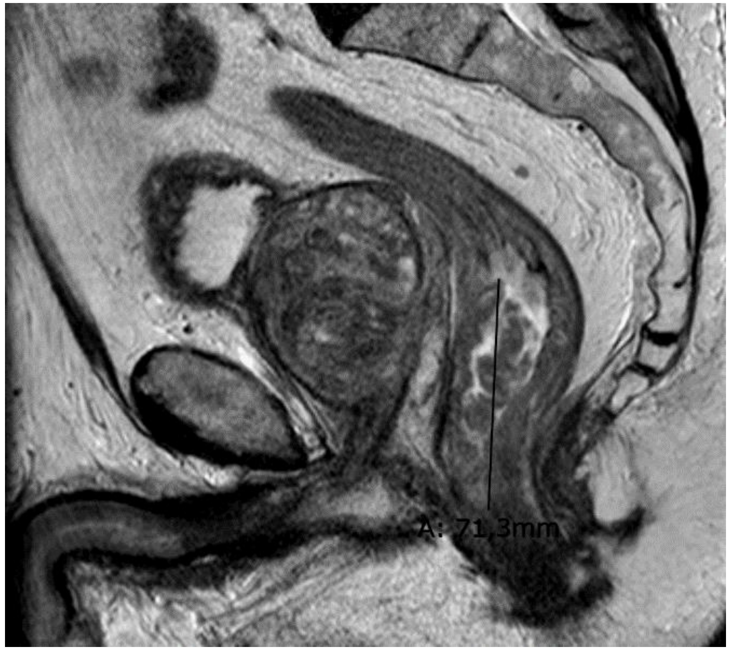

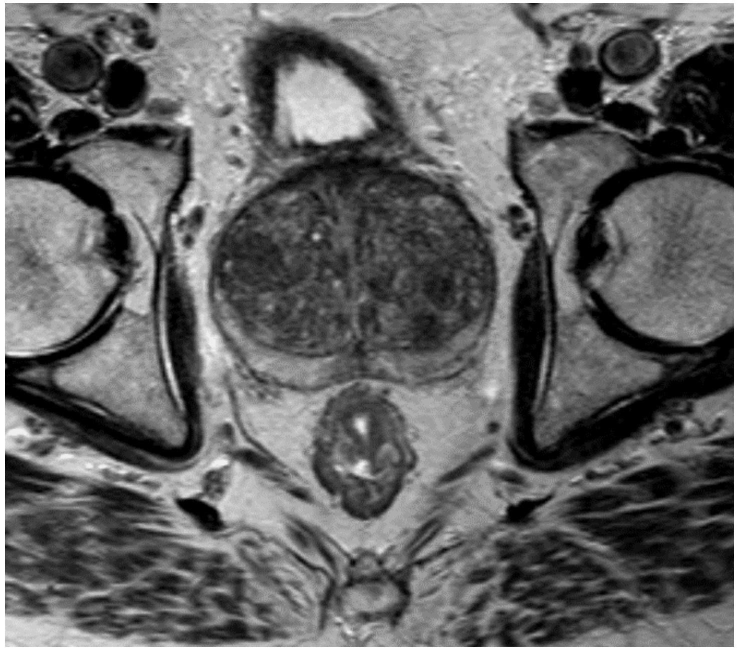

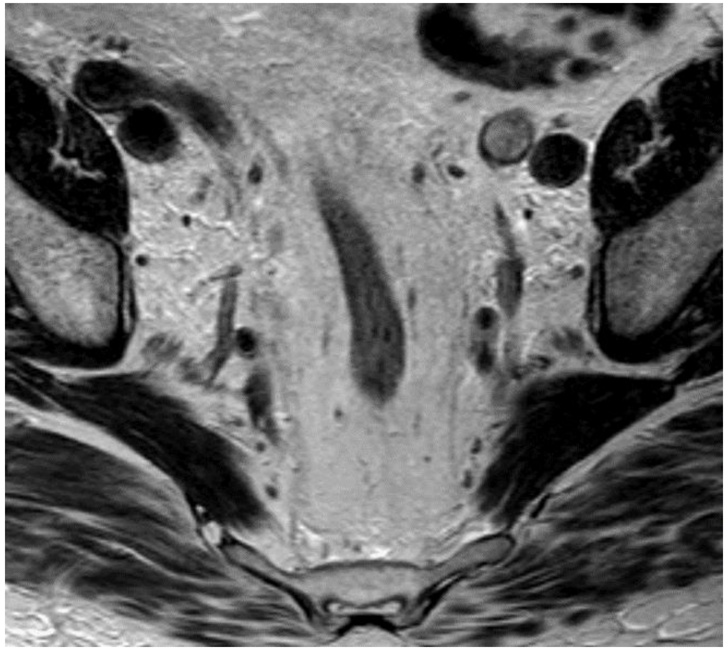

Patient 7

Snapshot

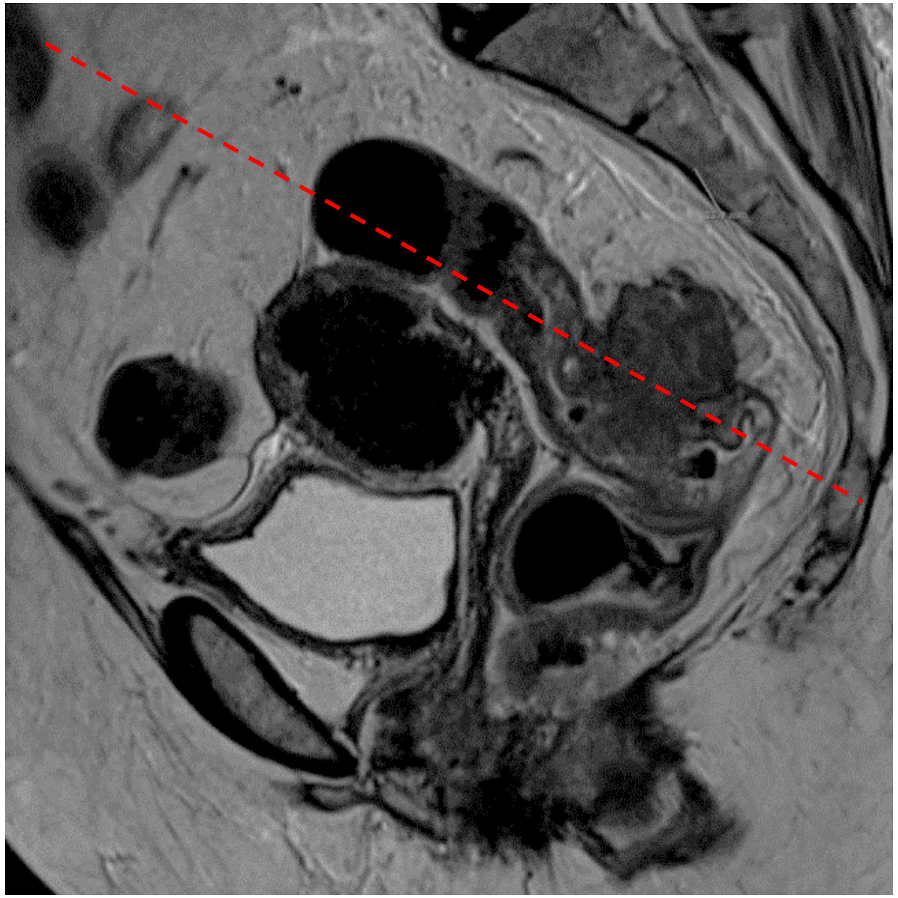

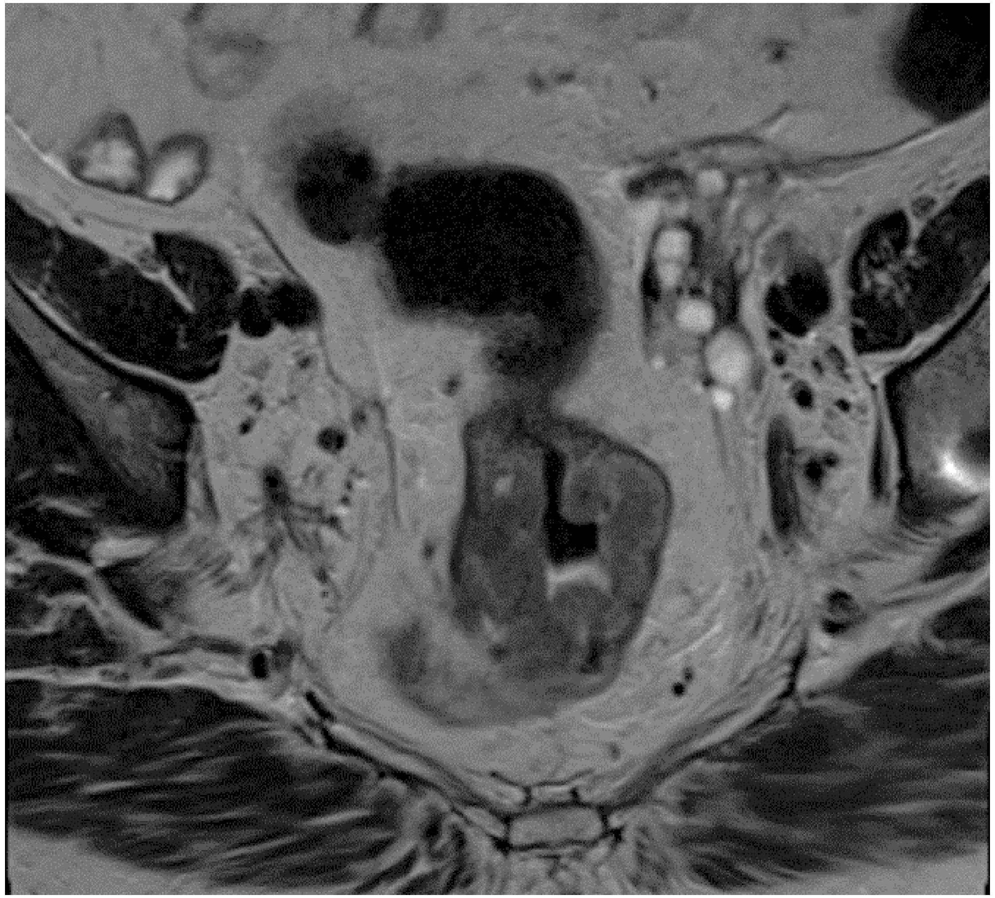

Patient 8

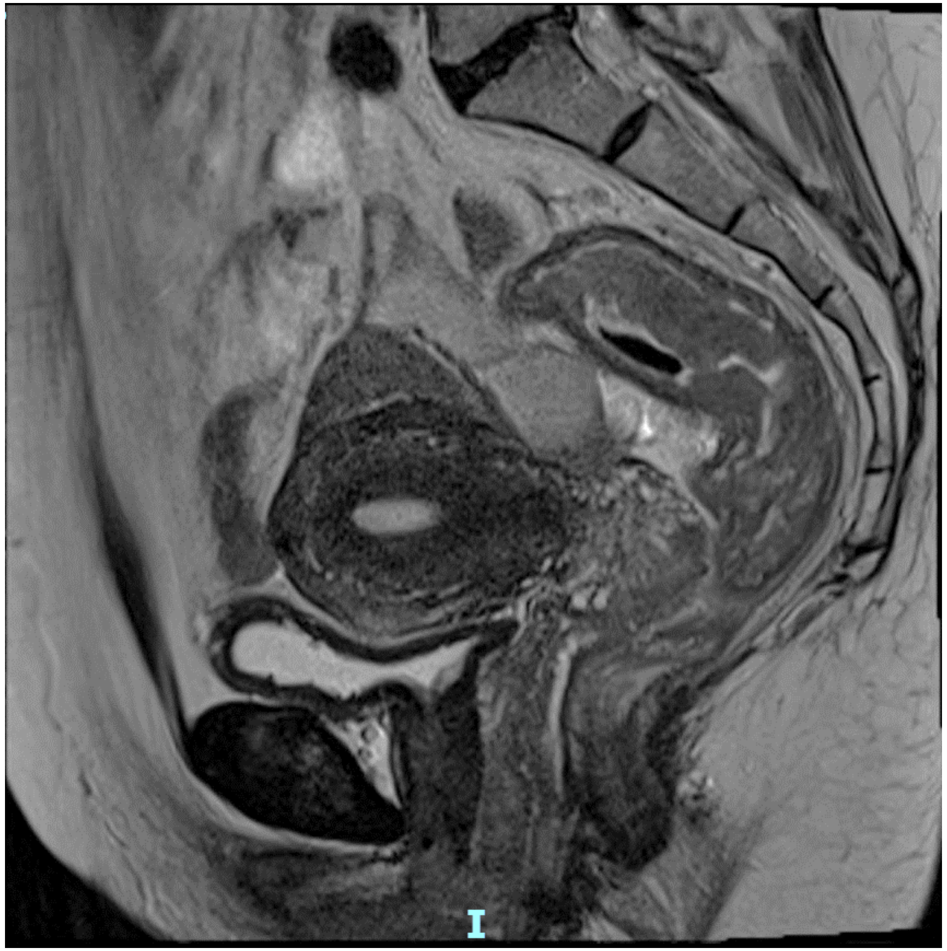

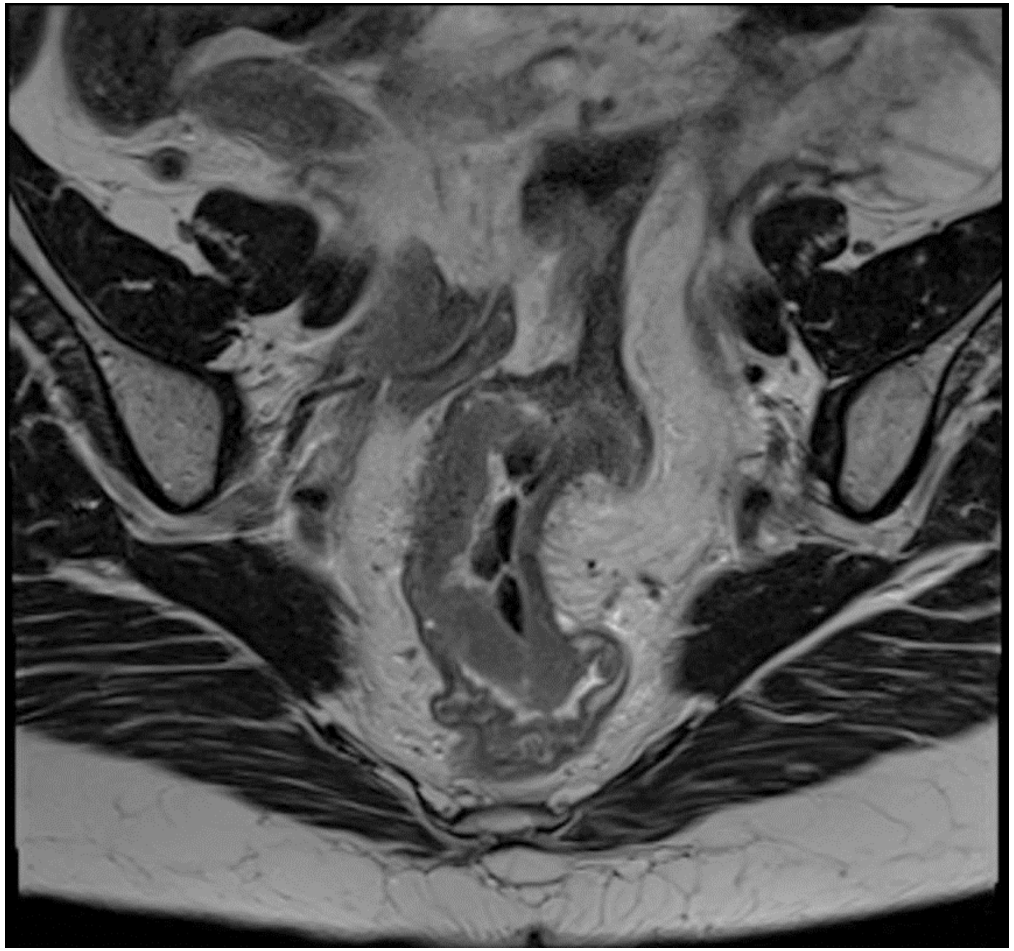

Patient 9

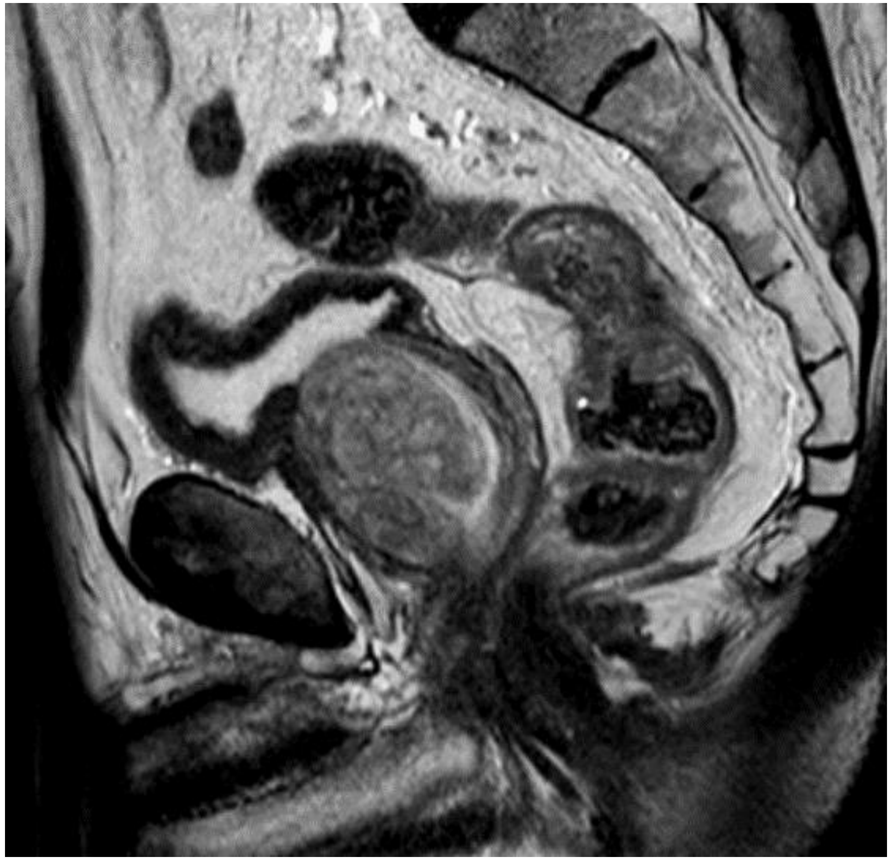

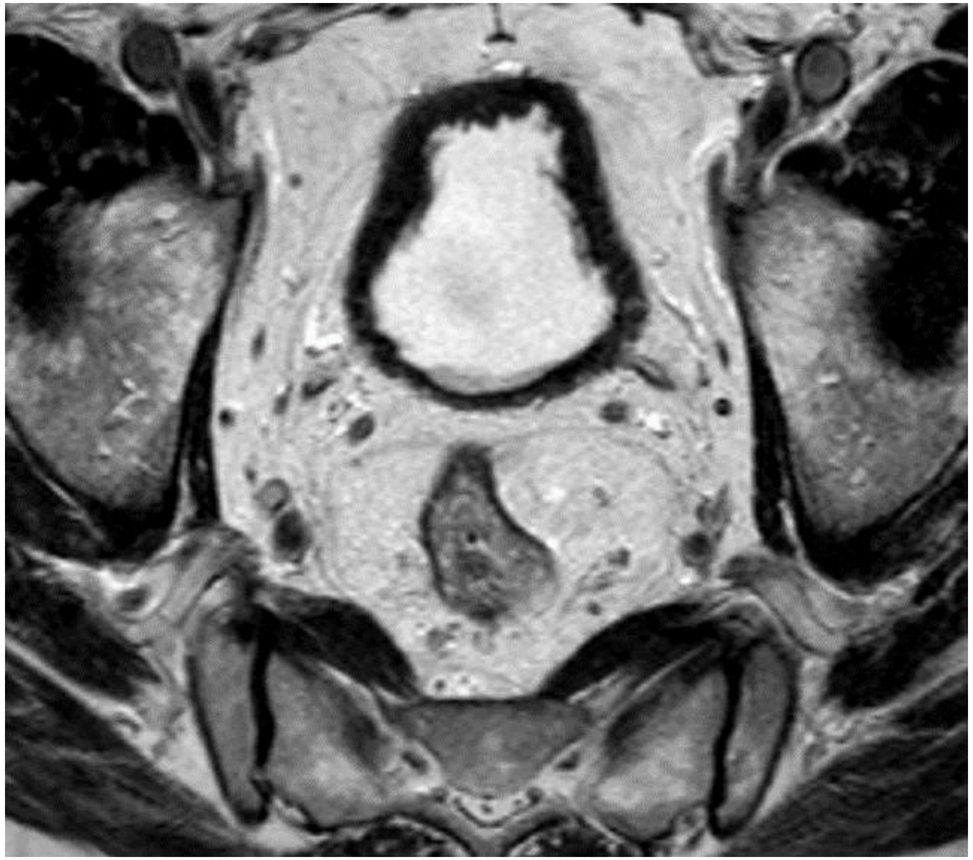

Patient 10

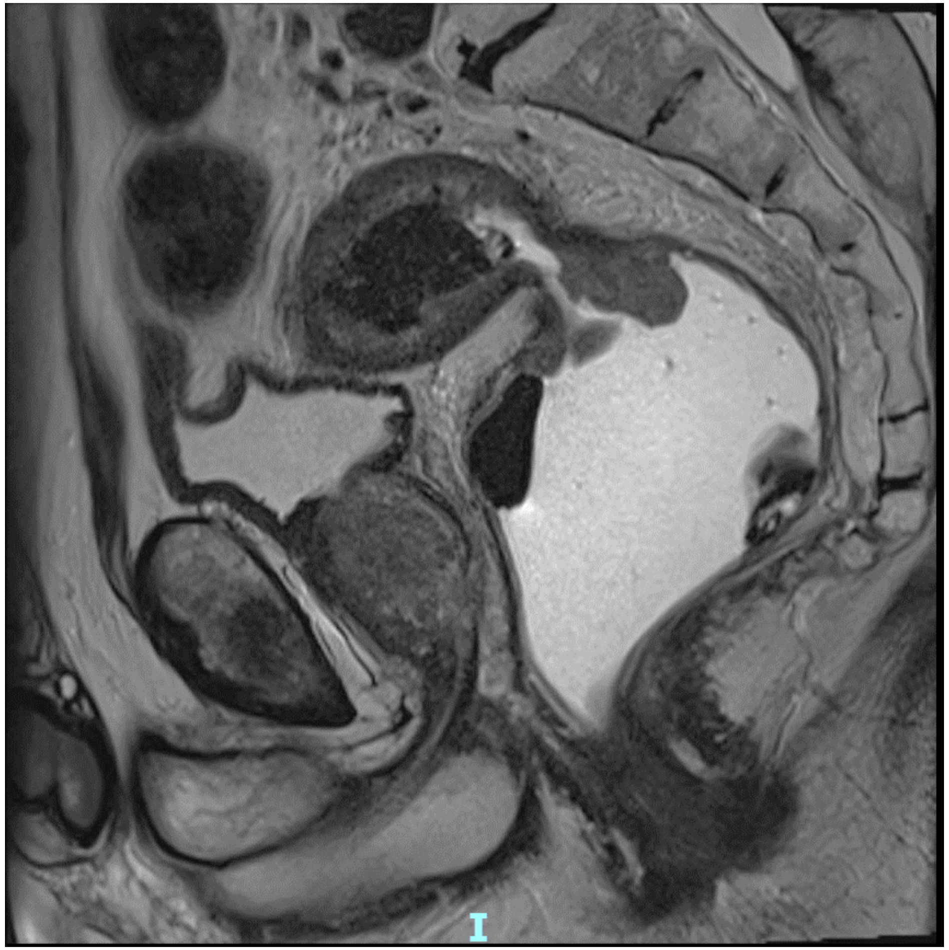

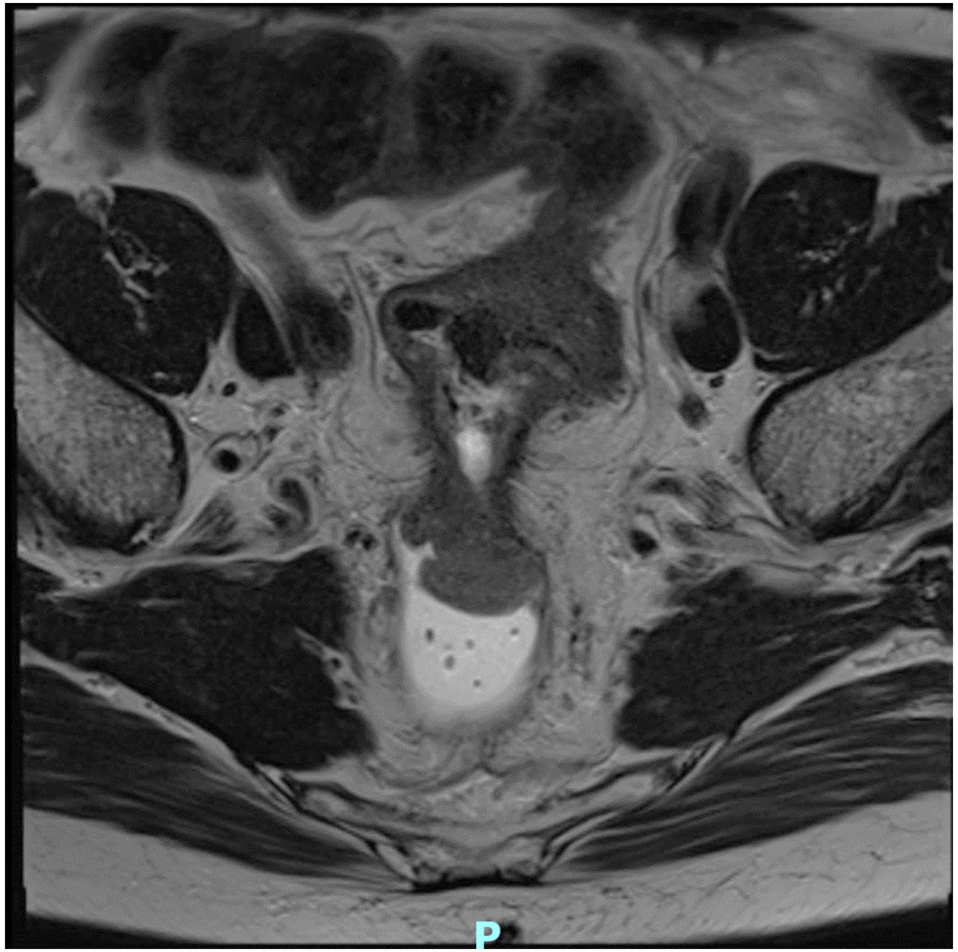

Patient 11

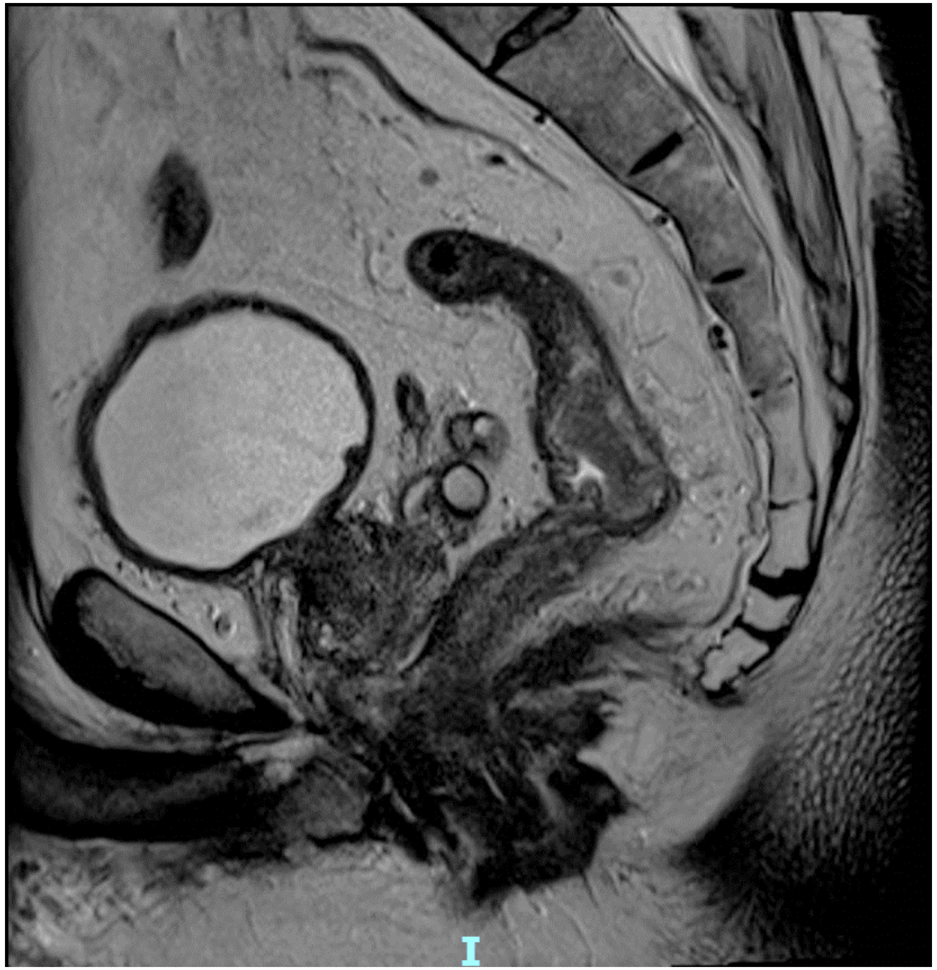

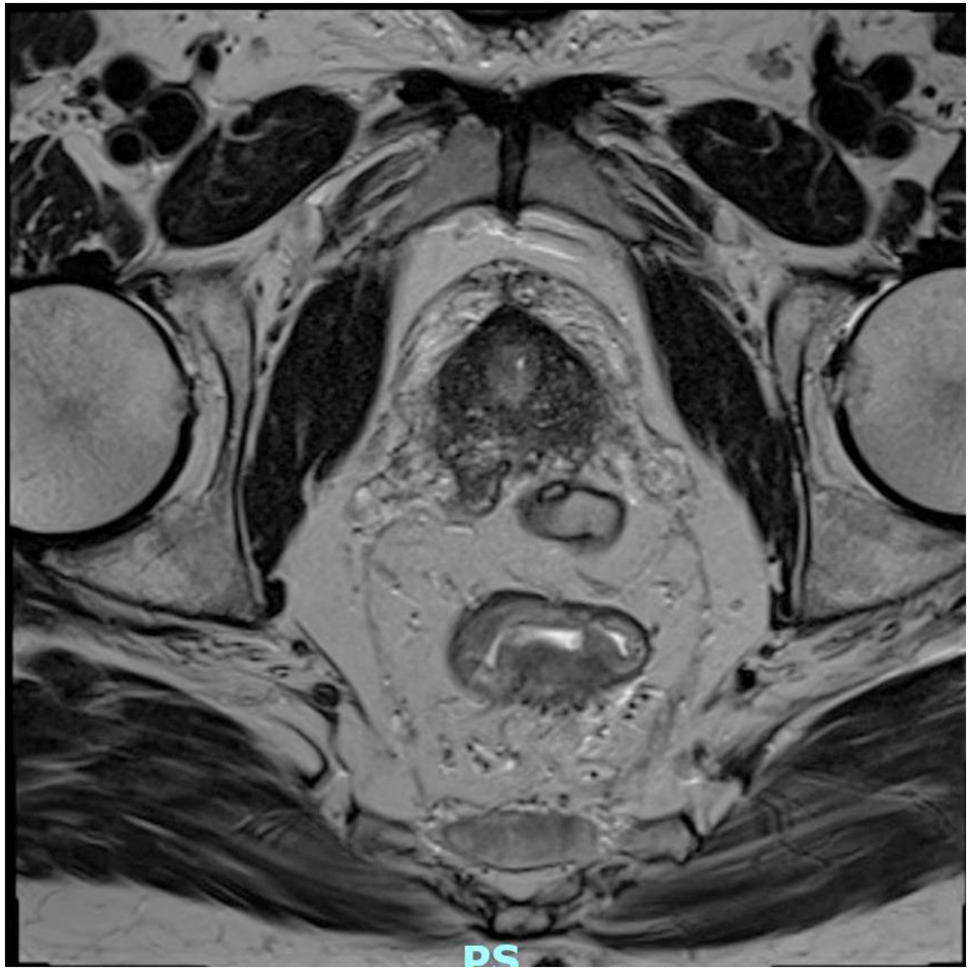

Patient 12

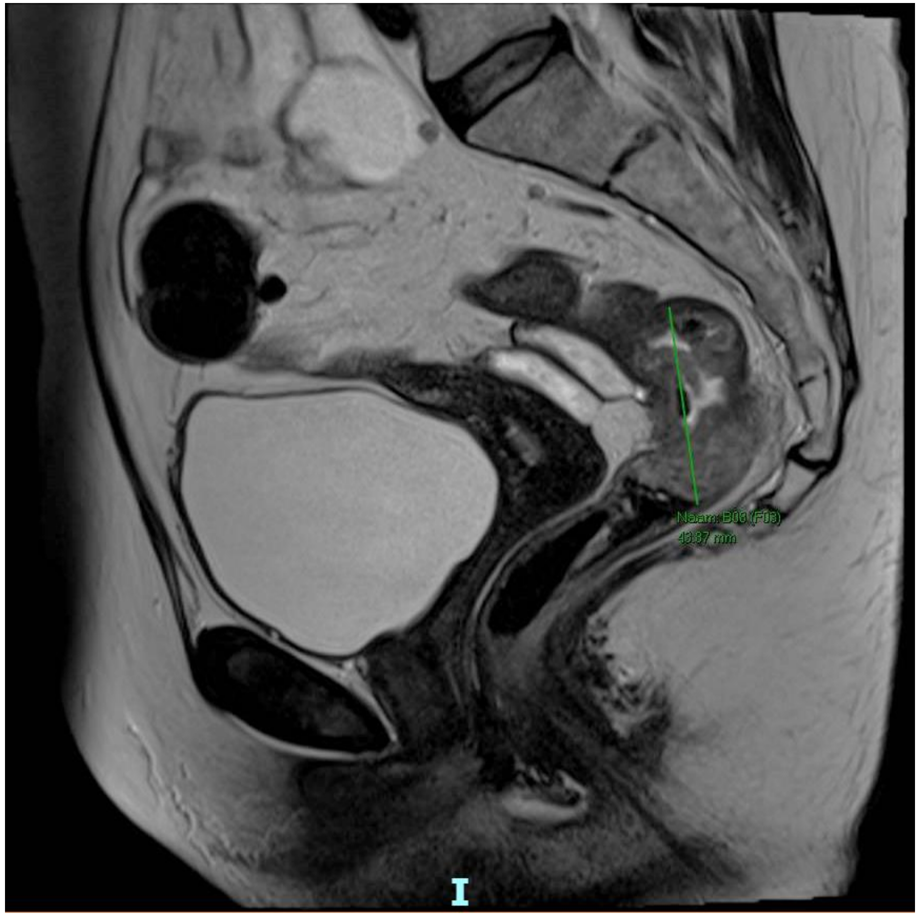

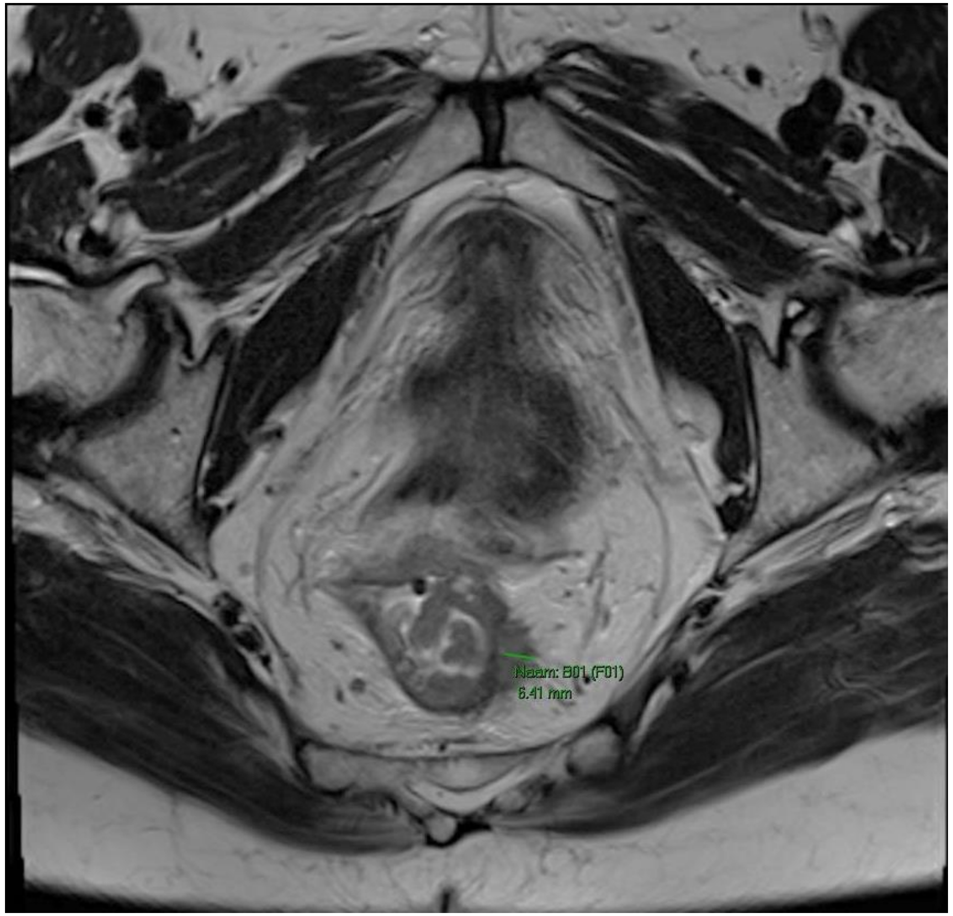

Patient 13

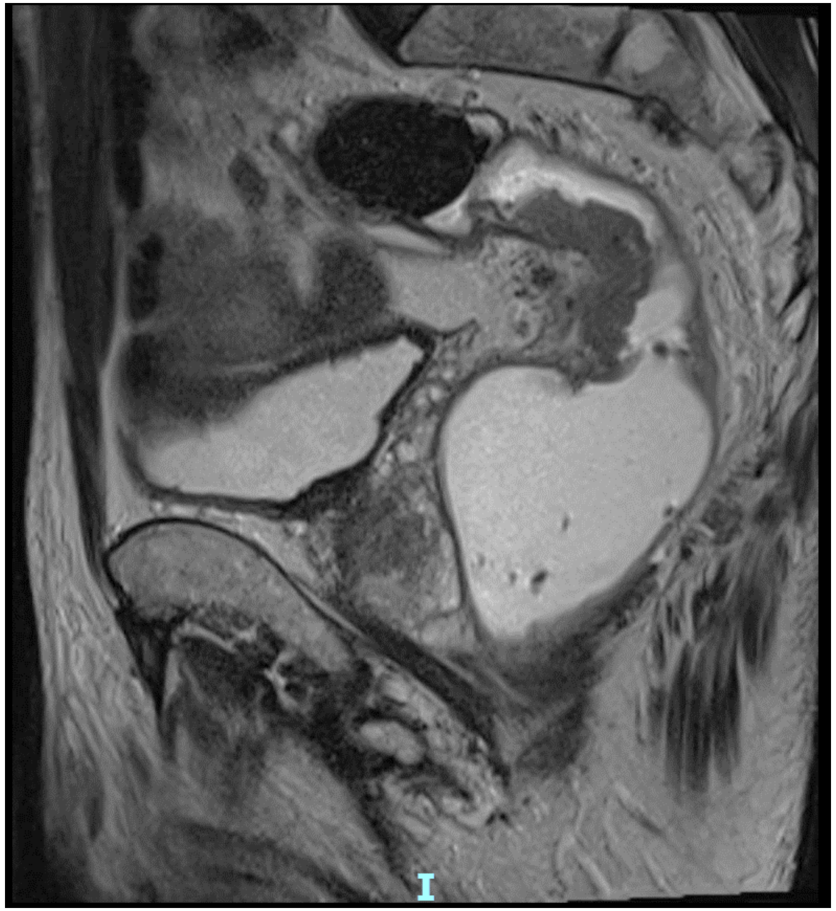

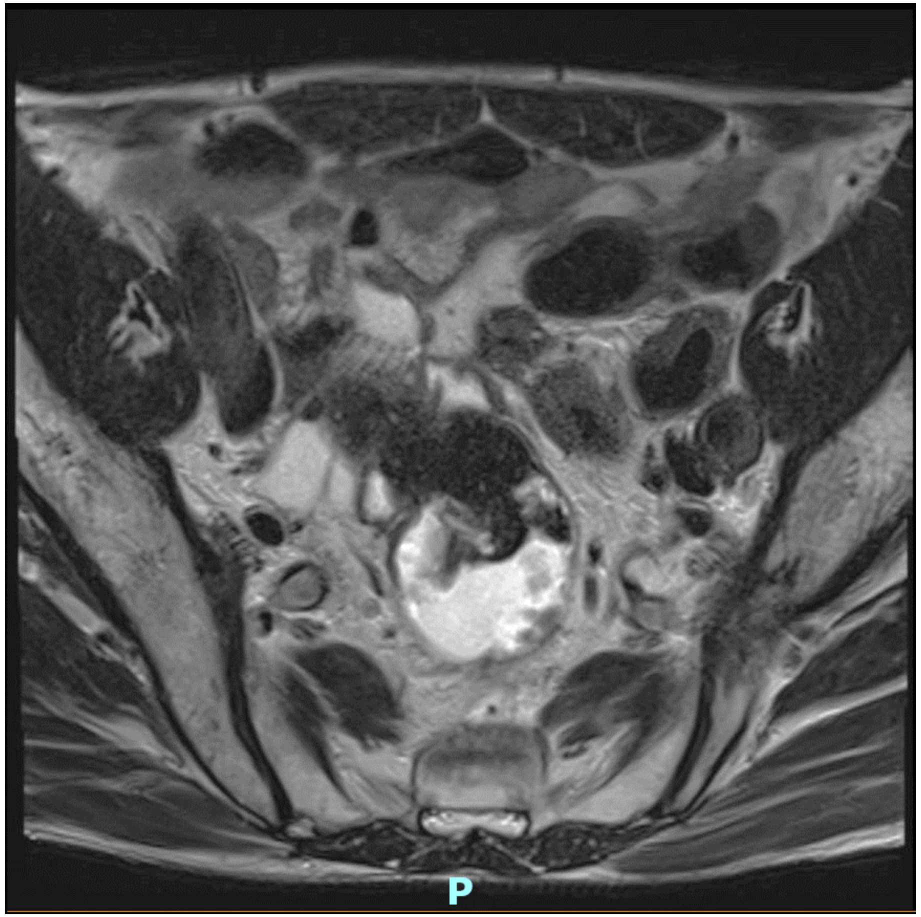

Patient 14

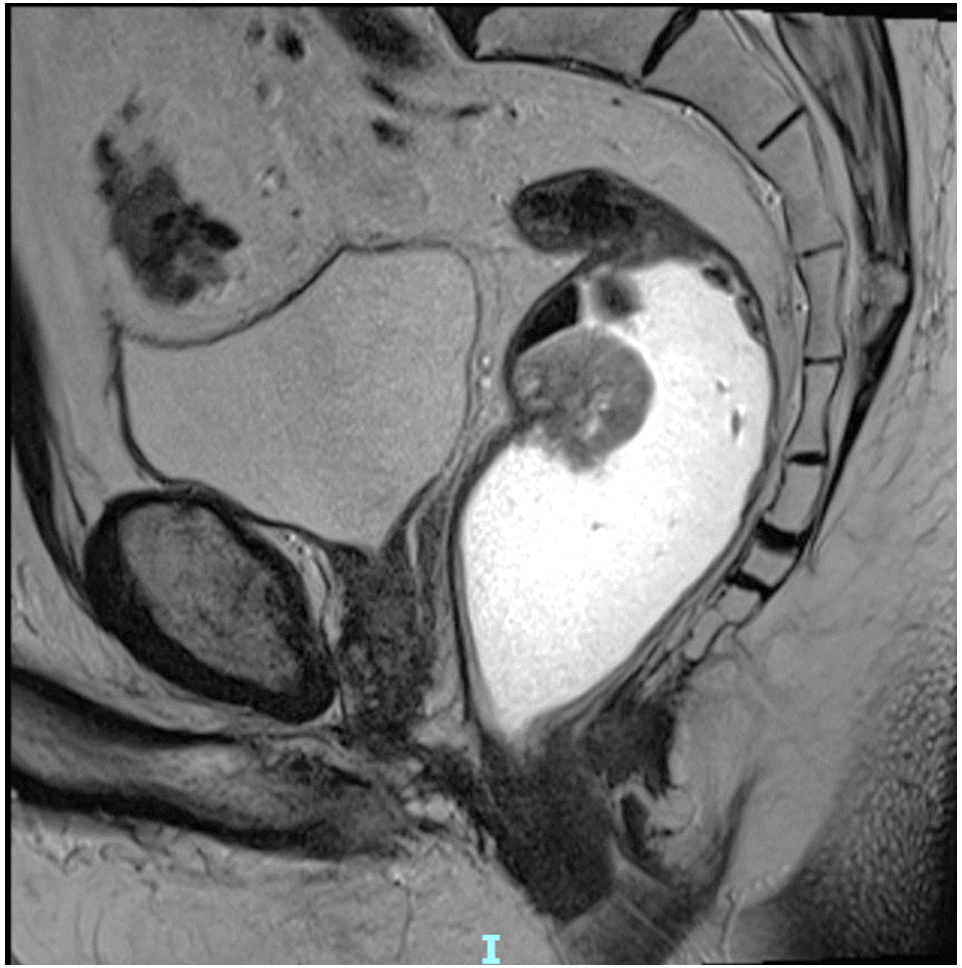

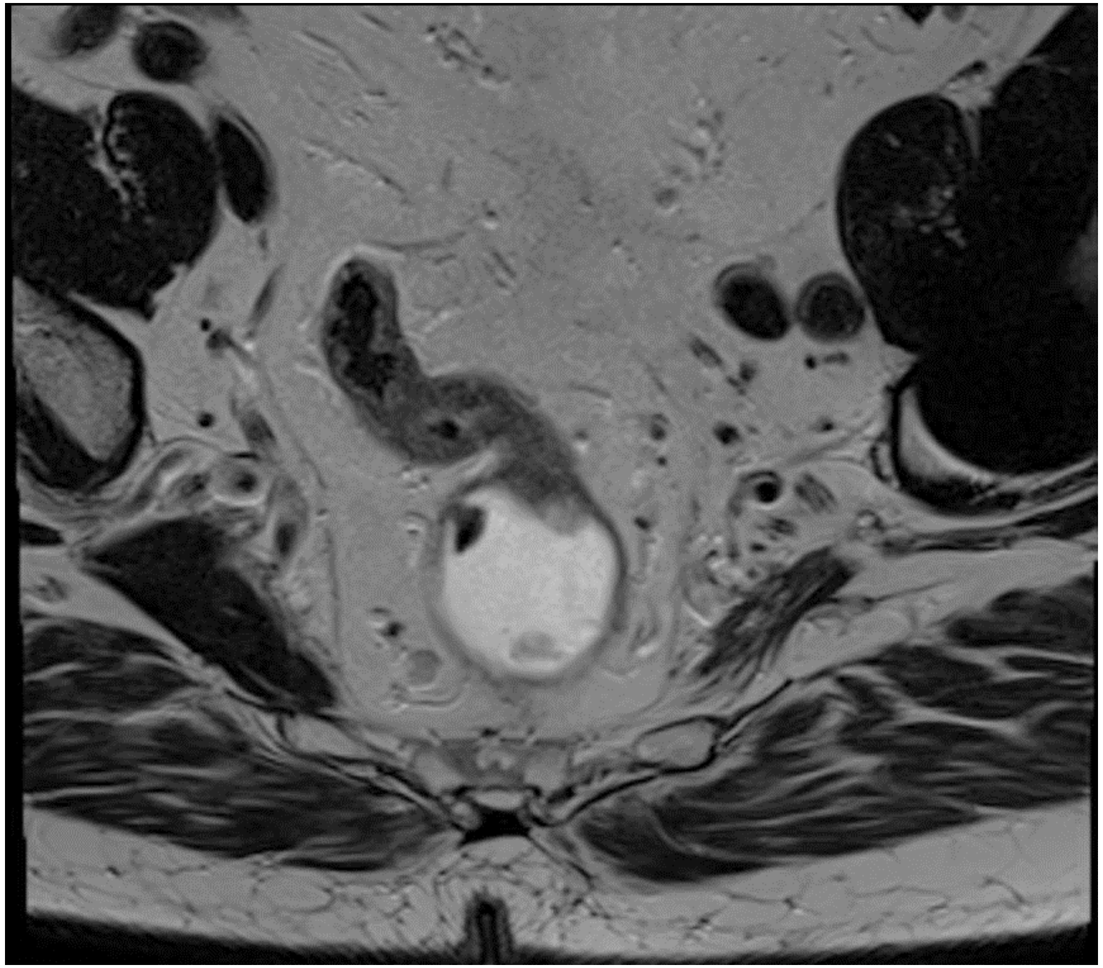

Patient 15

Snapshot

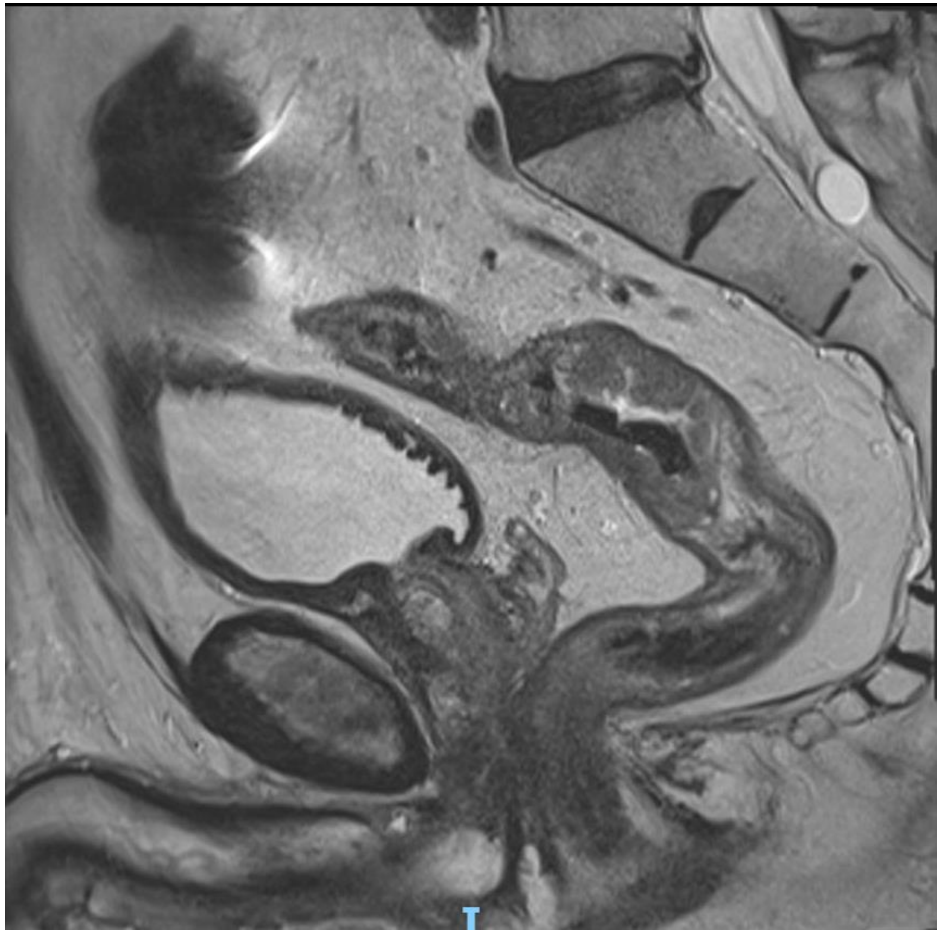

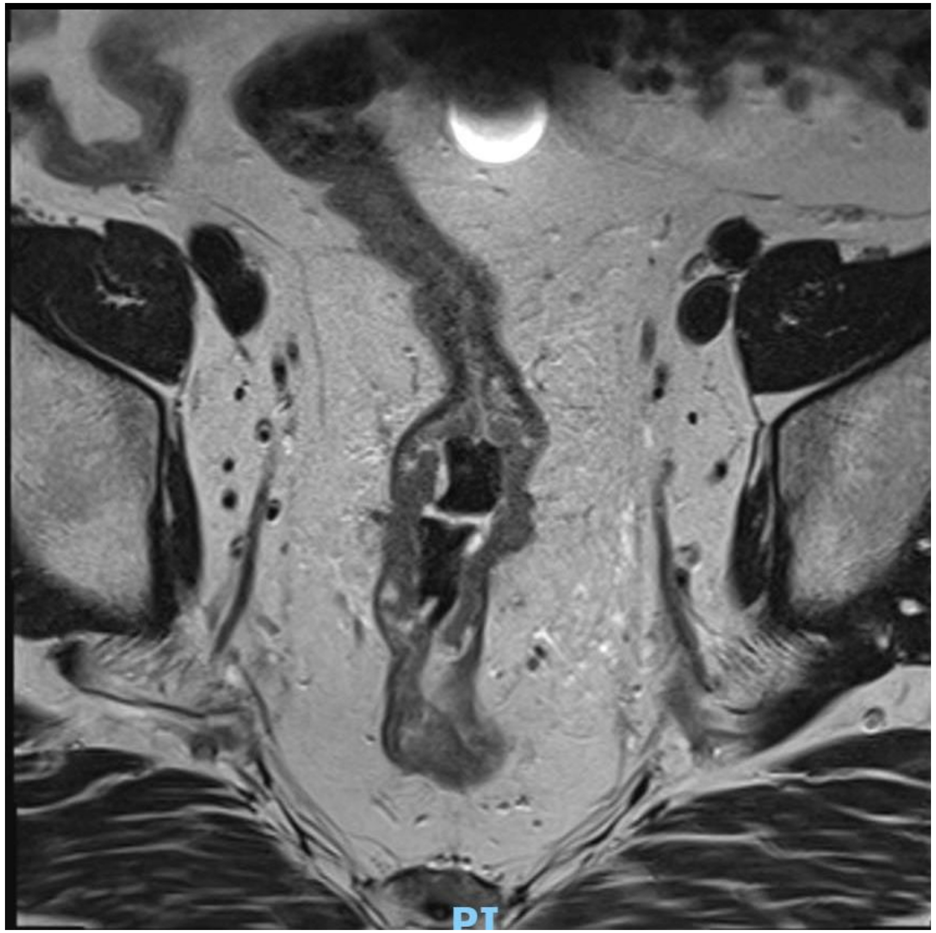

Patient 16

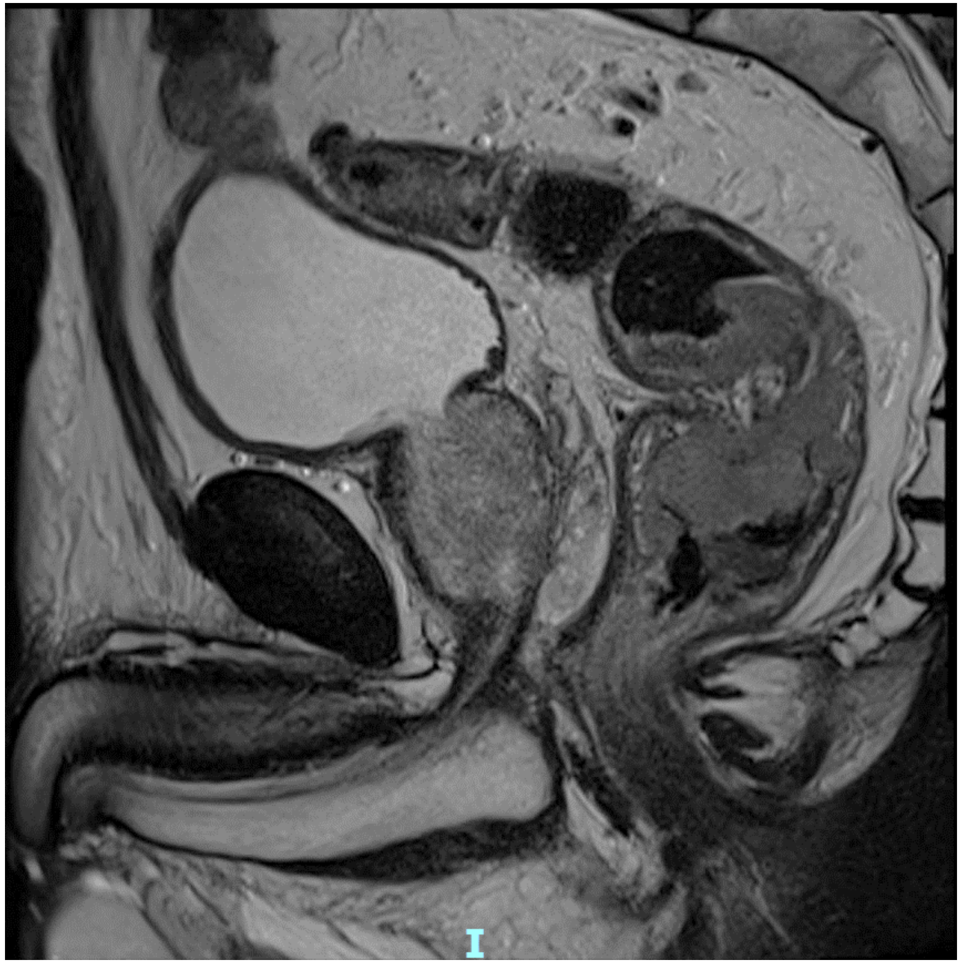

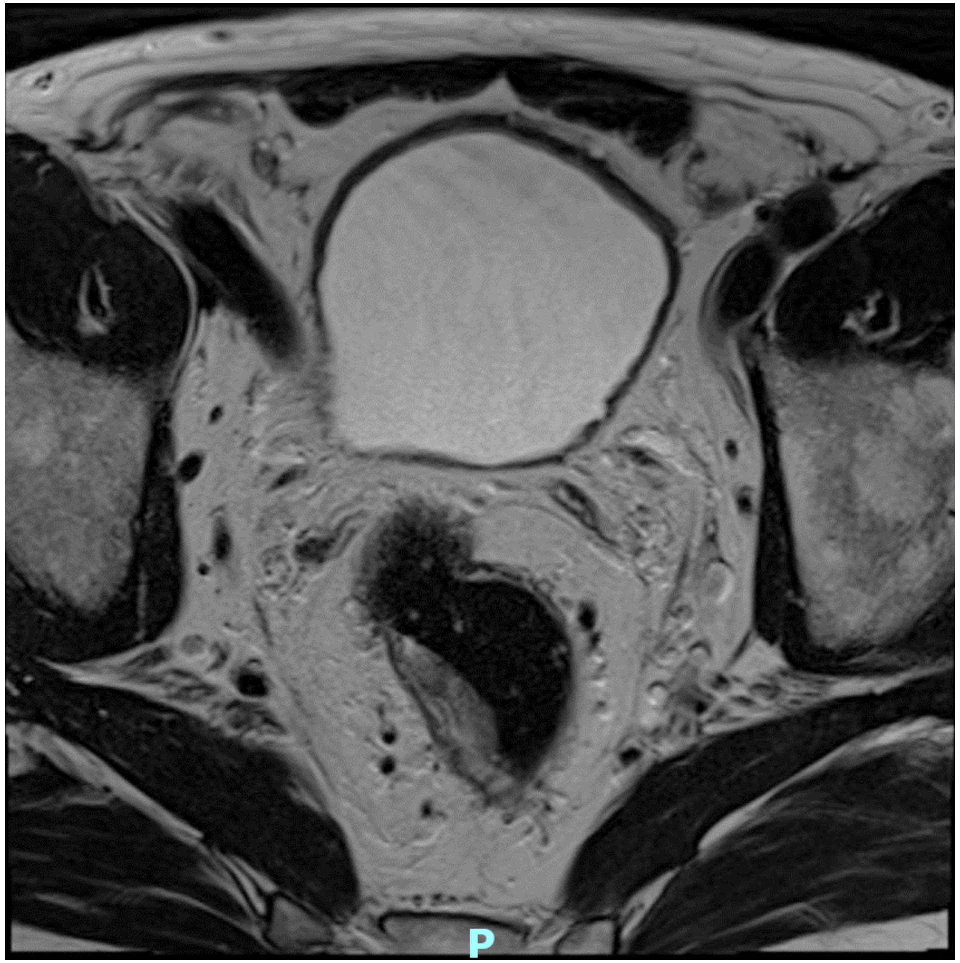

Patient 17

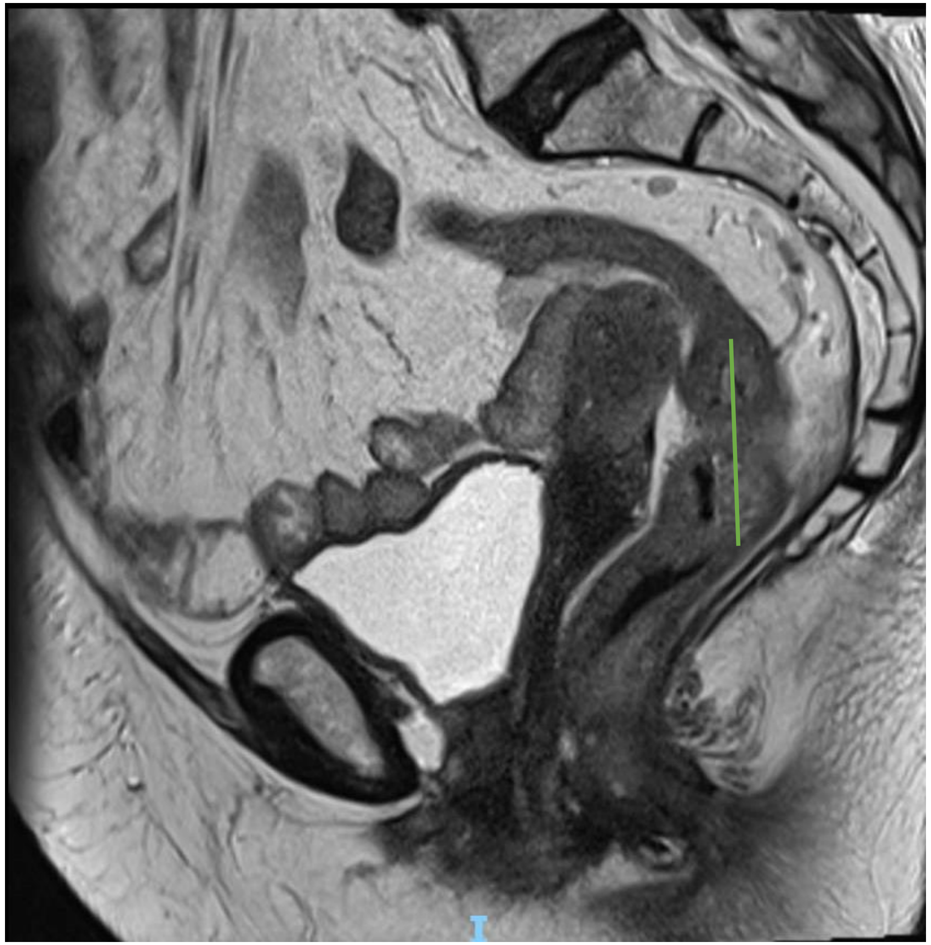

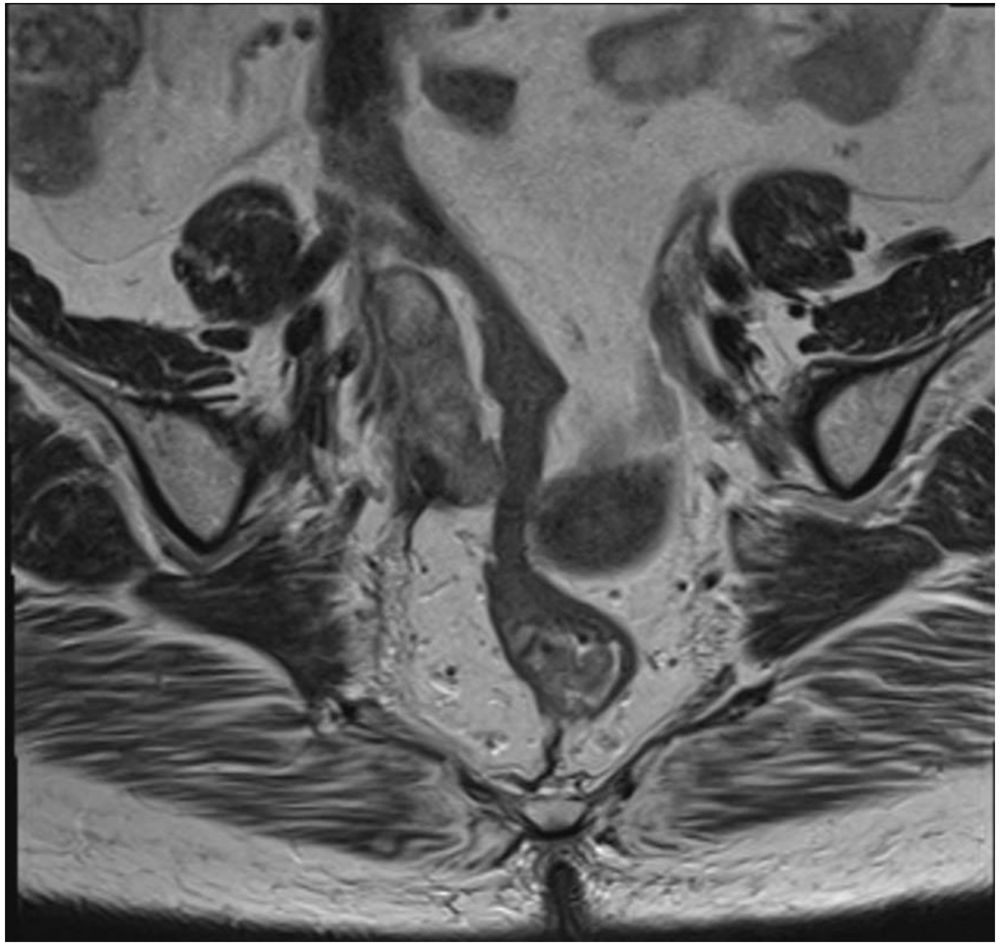

Patient 18

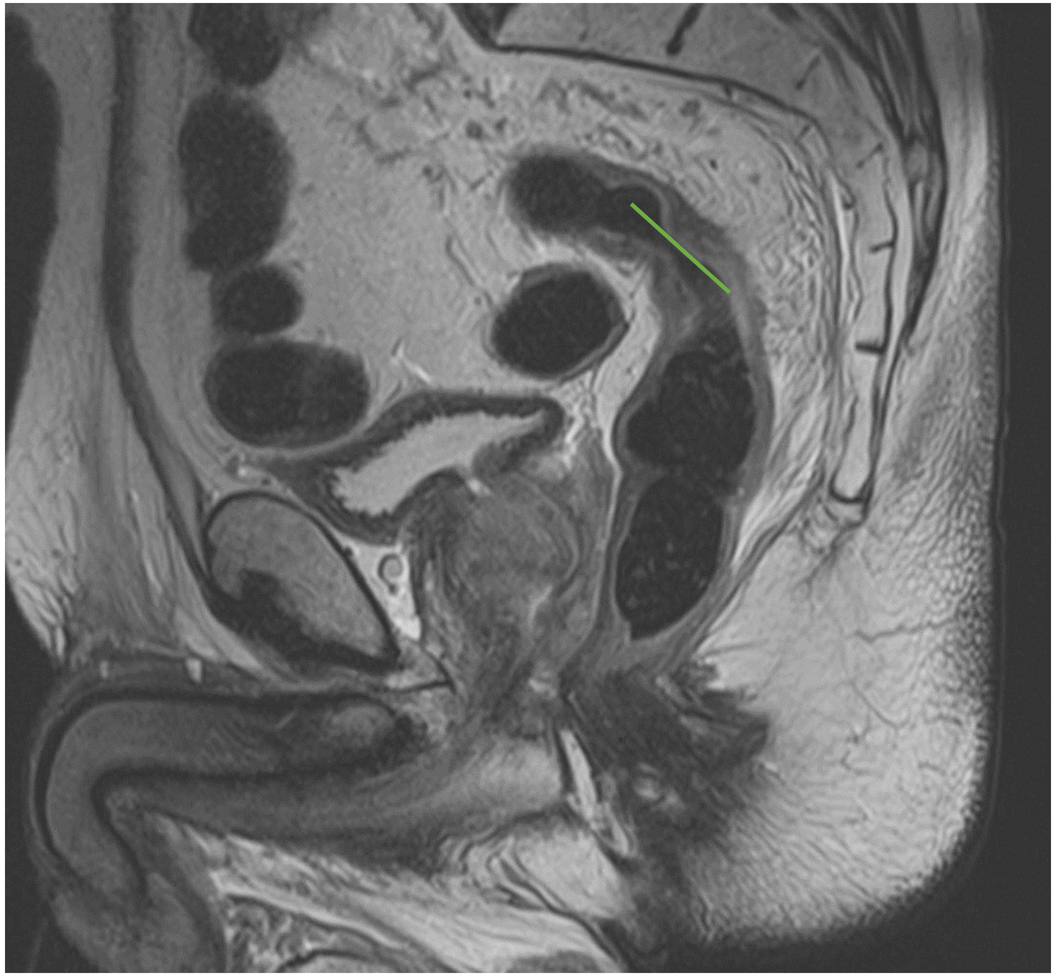

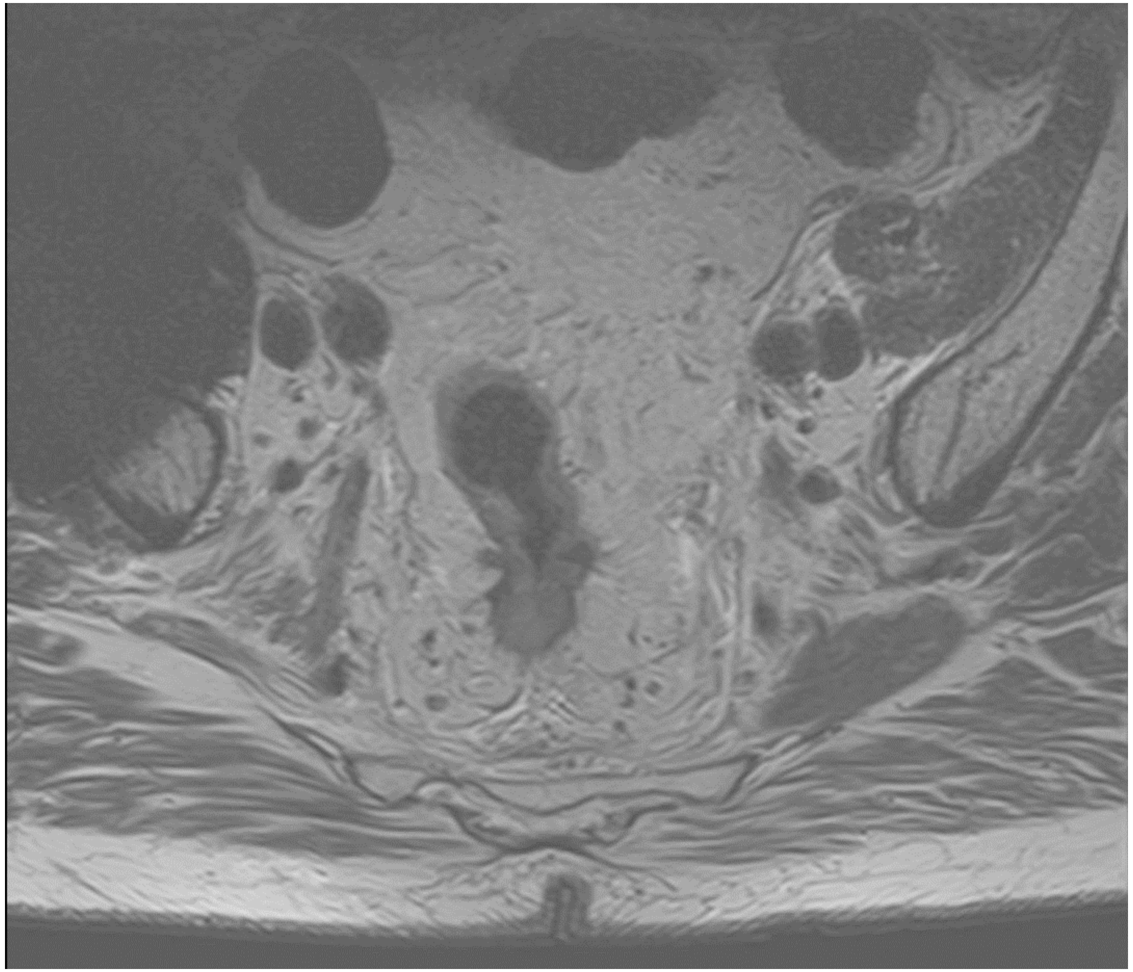

Patient 19

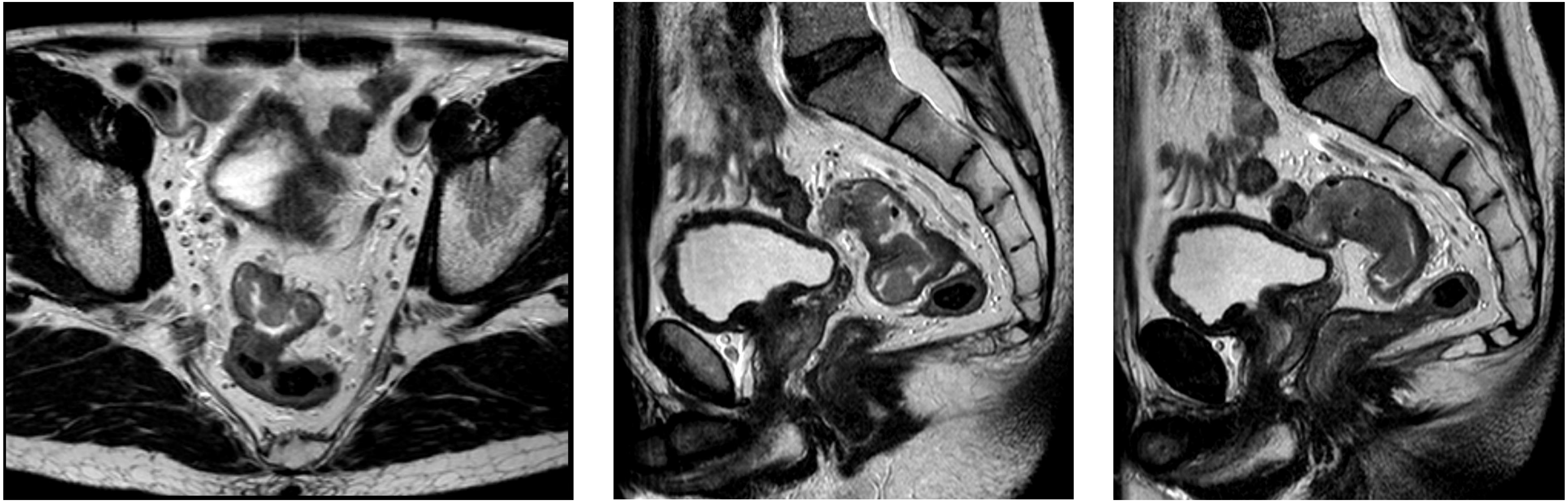

Patient 20

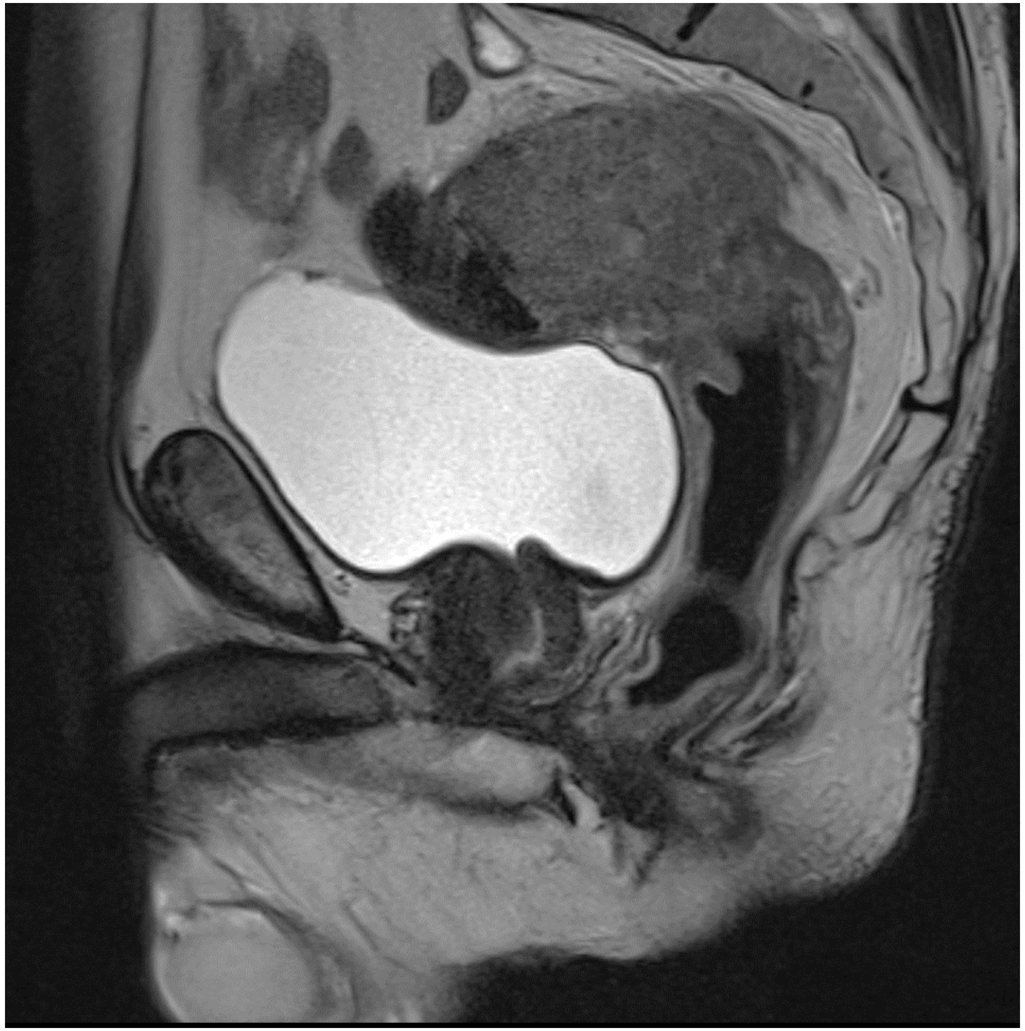

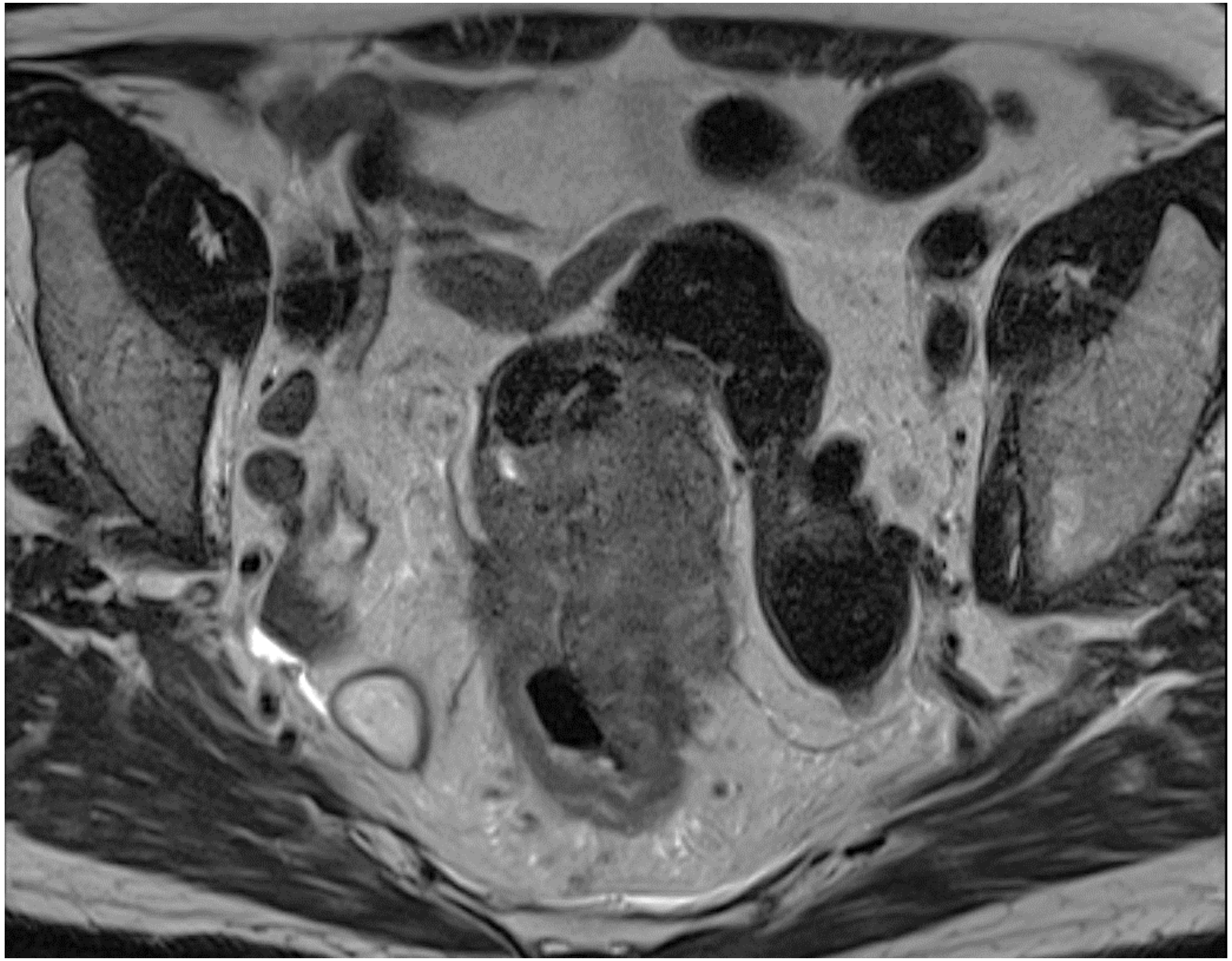

Supplement: Supplementary file 2 — Supplementary file2 (PDF 6866 KB) [file 10151_2023_2803_MOESM2_ESM.pdf]
